# Myeloid cell-expressed MNDA enhances M2 polarization to facilitate the metastasis of hepatocellular carcinoma

**DOI:** 10.7150/ijbs.91877

**Published:** 2024-05-05

**Authors:** Yanru Meng, Mengxin Zhang, Xinli Li, Xinxin Wang, Qian Dong, Hu Zhang, Yuanjun Zhai, Qin Song, Fuchu He, Chunyan Tian, Aihua Sun

**Affiliations:** 1 Department of Immunology, Medical College of Qingdao University, Qingdao, Shandong 266071, China.; 2 State Key Laboratory of Proteomics, National Center for Protein Sciences (Beijing), Beijing Proteome Research Center, Beijing Institute of Lifeomics, Beijing 102206, China.; 3 Research Unit of Proteomics Dirven Cancer Precision Medicine, Chinese Academy of Medical Sciences, Beijing, China.; 4 Department of Pathology, Beijing You'an Hospital, Capital Medical University, Beijing 100069, China.; 5 College of Life Sciences, Hebei University, Baoding 071002, Hebei, China.

**Keywords:** MNDA, HCC, metastasis, M2 polarization, exosome

## Abstract

Stable infiltration of myeloid cells, especially tumor-associated M2 macrophages, acts as one of the essential features of the tumor immune microenvironment by promoting the malignant progression of hepatocellular carcinoma (HCC). However, the factors affecting the infiltration of M2 macrophages are not fully understood. In this study, we found the molecular subtypes of HCC with the worst prognosis are characterized by immune disorders dominated by myeloid cell infiltration. Myeloid cell nuclear differentiation antigen (MNDA) was significantly elevated in the most aggressive subtype and exhibited a positively correlation with M2 infiltration and HCC metastasis. Moreover, MNDA functioned as an independent prognostic predictor and has a good synergistic effect with some existing prognostic clinical indicators. We further confirmed that MNDA was primarily expressed in tumor M2 macrophages and contributed to the enhancement of its polarization by upregulating the expression of the M2 polarization enhancers. Furthermore, MNDA could drive the secretion of M2 macrophage-derived pro-metastasis proteins to accelerate HCC cells metastasis both *in vivo* and *in vitro*. In summary, MNDA exerts a protumor role by promoting M2 macrophages polarization and HCC metastasis, and can serve as a potential biomarker and therapeutic target for HCC.

## Background

Liver cancer ranks as the sixth most common cancer in the world and is the third leading cause of tumor-associated death, and HCC accounts for 90% of cases. Despite significant advancements in traditional treatments such as surgery, intervention, and radiation therapy, as well as innovative approaches like targeted therapy and immunotherapy, the management of HCC still faces formidable challenges in terms of prevention, early detection, and limited treatment targets 1, 2. Notably, HCC is a typical inflammation-related cancer, characterized by complex interactions between immune cells and cancer cells within the HCC microenvironment 3. Targeted immunotherapy, despite high expectations, exhibits substantial variations in different patients, which can be attributed to the immune microenvironment's heterogeneity and a limited understanding of the specific immune molecular subtypes and driving events in HCC 4, 5.

Currently, the molecular classification of the immune microenvironment in HCC is being elucidated using multi-omics techniques. For example, by assessing inflammation-related gene expression, immune cell infiltration, and immune regulatory ligands, the immune class has been classified into two components: (1) Active Immune Response Subtype (∼65%) with overexpressed adaptive immune response genes, and (2) Exhausted Immune Response Subtype (∼35%) with immunosuppressive signals like TGF-ß and M2 macrophages 6. Using 10x Genomics and SMART-seq2 single-cell RNA sequencing, immune cells from various HCC tissues were extensively characterized. Two distinct states of macrophages have been identified in HCC tumors: TAM-like and MDSC-like states. Survival analysis links the characteristic genes of TAM-like state to poor prognosis, including the expression of two key genes, SLC40A1 and GPNMB, which are also associated with unfavorable prognosis 7. From a proteomic perspective, we have previously investigated the heterogeneity in early-stage hepatocellular carcinoma and stratified the cohort into the subtypes S-I, S-II and S-III. Patients with HCC of the S-III subtype have a higher level of immune infiltration (especially myeloid cells, exhausted T cells, etc.), the lowest overall survival rate and the greatest risk of a poor prognosis after first-line surgery 8. However, there is a scarcity of effective immune-related biomarkers and interpretive tools to guide clinical decision-making.

Tumor-associated macrophages (TAMs) plays a vital role in tumor microenvironment to facilitate tumor progression 9, 10. Generally, macrophages can be polarized to classical activation state (M1) and the alternative activation state (M2). M1 macrophages are activated by lipopolysaccharides (LPS) and interferon-gamma (IFN-γ) to produce proinflammatory cytokines, such as IL6, CXCL10 etc. However, TAMs are thought to resemble M2 macrophages, which are activated by IL-4, IL-10, IL-13 and produce IL-10, CCL22 etc. Recently, it has been reported that M2 macrophages promote tumor development through exosome-mediated communication with tumor cells 11, 12. Particularly, TAMs highly infiltrate S-III subtype of HCC tissues compared to other subtypes and are positively associated with relapse-free survival rate. Thus, delving into additional biomarkers and therapeutic targets connected to TAM and uncovering their association with HCC would greatly enhance their practical implementation in clinical settings.

Myeloid cell nuclear differentiation antigen (MNDA) is a member of the hematopoietic interferon-inducible nuclear proteins containing a 200-amino-acid repeat (HIN-200) family, which can induce cell cycle arrest, apoptosis, senescence, inflammation and recognize foreign double-stranded DNA 13-16. It has been reported that MNDA restricts HIV-1 and other viral pathogens by interfering with Sp1-dependent gene expression and supports an important role in innate antiviral immunity 17. Unlike other HIN-200 factors, MNDA is essential for IFNα induction by regulating the expression of IRF7, rather than acting as a cytosolic PRR for dsDNA 18. Moreover, MNDA has become a potential therapeutic target for sepsis and inflammatory pathologies by promoting the apoptosis of neutrophils 19. MNDA can effectively inhibit proliferation, induce apoptosis and reduce migration of osteosarcoma cells 20. Meanwhile, high expression of MNDA is associated with good overall survival in lung adenocarcinoma 21. Nevertheless, the functional and mechanistic roles of MNDA in HCC are not yet understood.

In this study, we demonstrated for the first time that MNDA exhibited significantly increased expression in the most aggressive subtype of HCC and acted as an independent prognostic predictor, showing a correlation with myeloid immune response and HCC metastasis. Furthermore, MNDA promoted HCC metastasis through enhancing the polarization of M2 macrophages and the secretion of pro-metastatic proteins partially via the exosome pathway. Thus, our study uncovered MNDA as a novel biomarker of poor prognosis in TMEs and might be exploited as a new strategy to reprogram M2 macrophages with the aim of reducing progression of HCC.

## Methods

### Public data acquisition

After acquiring the TCGA dataset of Liver Hepatocellular Carcinoma (LIHC) from UCSC Xena (https://xenabrowser.net/), we conducted data preprocessing by removing samples with incomplete survival information. Following this process, 307 tumor samples were retained for subsequent survival analysis. We gathered two distinct sets of Hepatocellular Carcinoma (HCC) proteomic datasets, accompanied by relevant clinical information, from published literature. These datasets are referred to as "Jiang et al's Cohort" 8 and "Gao et al's Cohort" 22. Jiang et al's Cohort comprises 101 tumor samples, 98 paracancerous samples, and Gao et al's Cohort comprises 159 pairs of tumor and paracancerous samples. In Jiang et al's Cohort, any missing values were addressed by imputing the minimum observed value. Furthermore, to facilitate subsequent analytical procedures, log2 transformation was applied to the data. The scRNA-seq data and the data analysis result, such as dimension reduction, celltype annotation etc. were downloaded from the TISCH portal (http://tisch.comp-genomics.org/). The GSE140228_10X dataset contains 62530 cells from 5 HCC patients, 7776 of which were identified as macrophages/monocytes.

### Difference analysis

The Welch's t-test was employed to analyze the distinctions between the two group. The log-fold change (logFC) is computed by taking the logarithm of the ratio between the means of each group. And p-values were adjusted by FDR (q-value). The screening criteria for specific highly expressed proteins of the S-III/S-Pf subtype in the cohort's proteomics dataset are as follows: p value is less than 0.05 and logFC(S-III/S-I) and logFC(S-III/S-II) is greater than 2 in Jiang et al's cohort; p value is less than 0.05 and logFC(S-Pf/S-Mb) and logFC(S-Pf/S-Me) is greater than 1 in Jiang et al's cohort. It seems that different filtering conditions are applied due to variations in the quantitative methods used in the two data sets. For the RNA-seq data of Ctrl and MNDA knockdown cell samples, significantly differentially expressed genes were considered following the criteria: FDR less than 0.05; logFC(Ctrl/shMDNA) greater than 1 (Supplementary Table 3). To screen proteins potentially involved in the secretion of MNDA, the following screening conditions are applied in exosome proteomics data: p value less than 0.05; logFC(Ctrl/shMDNA) greater than 0 (Supplementary Table 4).

### Survival analysis

Kaplan-Meier plots (Log-rank test) were used to describe the overall survival. The survival curves were calculated with the R function survfit from the R package survivall (version 3.5-0) with the formula Surv (time, vitalstatus)~categorie and plotted with the R function ggsurvplot from the R package survminer (version 0.4.9). The surv_cutpoint function from the survminer package is utilized to determine the optimal cut-off value. The main principle behind this method is the Maximally Selected LogRank statistics, which involves grouping each value of protein abundance separately and calculating the survival difference between the two groups. The cut-off value with the largest difference in survival is identified as the optimal cut-off value. This approach helps to determine the most suitable threshold for stratifying patients based on protein abundance levels, facilitating more accurate prognostic assessments in the context of survival analysis.

To demonstrate MNDA as an independent prognosticator, univariate and multivariate Cox regression analysis was performed for each clinical factor (age, gender, liver cirrhosis, AFP level, tumor number, diameter of tumor, BCLC) in R software. Subsequently, the power of the "coxph" function was harnessed to construct a Cox Regression model and compute Hazard Ratios (HR). A result of HR > 1 and p value < 0.05 indicates an increased risk of the event, A result of HR < 1 and p value < 0.05 indicates a decreased risk, and a hazard ratio of 1 or p value ≥ 0.05 suggests no difference in risk between groups.

### GO biological process and KEGG pathway enrichment analysis

Pathway enrichment analysis was performed using Metascape Gene Annotation & Analysis Resource 23 that uses several ontology sources: KEGG Pathway, GO Biological Processes, Reactome Gene Sets, Canonical Pathways and CORUM (https://metascape.org/gp/index.html#/main/step1). The pathway with a significance of p.adjust < 0.05 were defined as significantly regulated. GSEA analysis was performed in R/Bioconductor package fgsea (version1.24.0) or clusterProfiler (version 4.6.0) referring to the inflammatory response and Liao metastasis pathway from the MSigDB database (http://www.gsea-msigdb.org/gsea/msigdb/index.jsp). Genesets overlapping at least ten genes with the DEPs and with a significance of p value < 0.05 were defined as significantly enriched. Heatmaps in this study were plotted using the R/Bioconductor package ComplexHeatmap (version 2.14.0).

### Gene set enrichment analysis (GSEA)

Gene set enrichment analysis (GSEA) is a method of analyzing and interpreting microarray and such data using biological knowledge. The core statistical method employed by GSEA is based on a non-parametric approach, particularly a rank-based statistical test. The data firstly generated an ordered list of logFC based on differential analysis, and then a predefined gene set receives an enrichment score (ES), which was a measure of statistical evidence rejecting the null hypothesis that its members were randomly distributed in the ordered list. The R package clusterProfiler was mainly used for GSEA analysis. And an adjusted P value < 0.05 was determined to be significant.

### Correlation analysis

Spearman correlation coefficient (rho) was applied to measure the correlation between MNDA and other proteins, the correlation between MNDA and M1/M2 macrophages score. The p-value corresponding to the correlation analysis was computed and adjusted by FDR correction, with an adjusted P value < 0.05 determined to be significantly positive. Correlation coefficient >0 means positive correlation and correlation coefficient <0 means negative correlation.

### Cell lines and cell culture

All hepatocellular carcinoma cell lines (Huh7, PLC/PRF/5 (PLC), MHCC-97H) were purchased from the Shanghai Cell Bank, Chinese Academy of Sciences (Shanghai, China), and were subjected to cell identification and mycoplasma detection. All hepatocellular carcinoma cell lines were cultured in DMEM (Gibco, USA) medium supplemented with 10% FBS and 1% penicillin-streptomycin (Gibco, USA). Human monocyte line THP-1 was purchased from Shanghai Zhongqiao Xinzhou Biotechnology Co., Ltd. and cell identification and mycoplasma detection were performed (Shanghai, China). The cell lines were cultured in RPMI-1640 (Gibco, USA) medium supplemented with 10% FBS and 1% penicillin of streptomycin (Gibco, USA). All cells were maintained in a humidified incubator at 37°C with 5% CO_2_ cells within 50 passages were used for experiments.

### Macrophage polarization assay

THP-1 cells were treated with 162 nM phorbol 12-myristate 13-acetate (PMA, P8139, Sigma) for 24 h to differentiate into M0 macrophages. On the basis of M0, cells were polarized to the M1 phenotype after treatment with 100 ng/mL Lipopolysaccharide (LPS, L2880, Sigma) and 20 ng/mL Interferon-γ (IFN-γ, 300-02, Peprotech) for 24 hours. Based on this M0 state, cells were polarized to the M2 phenotype after treatment with 20 ng/mL interleukin-4 (IL-4, 200-04, Peprotech) for 48 hours.

### MNDA Knockdown stable cell line generation

We used retroviral vectors containing short hairpin (sh) RNA for MNDA CCCAAACAGAATTATCGAAAT (denoted as shRNA1), GCACAATATCAAGTGTGAGAA (denoted as shRNA2), to transfect THP-1 cells. The transfected cells were selected in 4 μg/ml puromycin.

### Immunostaining

Liver cancer tissues were first harvested and fixed overnight in 4% paraformaldehyde (PFA). Thereafter, the tissues were embedded in paraffin and sliced at 5 µm. The sections were blocked with 10% goat serum for 30 min at 37°C and then incubated overnight at 4°C with the following primary antibodies: MNDA (HPA034532, 1:1000; sigma), CD163 (ab182422, 1:100; Abcam), CD3 (ab5690, 1:100; Abcam). Samples were then incubated with the corresponding Alexa Fluor-conjugated secondary antibodies (1:100, Thermo Fisher Scientific) and finally examined using a confocal microscope (LSM 880, Carl Zeiss AG).

### Isolation and characterization of exosomes

When macrophage confluence reached 80-90%, the cell culture medium was removed. Cells were washed twice with pre-warmed PBS and then macrophages were cultured in conditioned medium supplemented with 10% exosome-free fetal bovine serum (FBS, C3801-0050, VivaCell) for 48 hours. Approximately 50 mL of cell culture medium was collected for each cell line, the collected medium was centrifuged at 300 × g for 10 minutes, followed by centrifugation at 2,000 × g for 30 minutes to remove cell debris. Next, the supernatant was filtered using a 0.22-µm filter (Millipore). A 12,000 × g centrifugation was performed for 40 min, the precipitate was discarded, and the supernatant was retained. The exosomes in the supernatant were precipitated by ultracentrifugation at 100,000 × g for 90 min. After washing the exosome precipitate in PBS, the exosomes were precipitated again and resuspended in 500 μL of PBS. The protein content of the exosomes was determined by using the BCA Protein Assay Kit (Thermo Fisher Scientific). The exosomes were stored at -80°C for further use. Expression of exosome-specific marker proteins, such as CD63 and CD81, was detected by Western blotting. Exosomes were identified using transmission electron microscopy. First, 10 μL of exosomes were added dropwise to the copper grid, left for 1 min, and then the liquid was blotted from the side with filter paper. Next, 10 μL of uranyl acetate was added dropwise, left for 1 min, and then imaged at 100 kv for electron microscopic detection. The particle size and concentration of exosomes were measured using nanoparticle tracking analysis (NTA). First, frozen samples were taken, thawed in a 25°C water bath, and placed on ice. Then, exosome samples were diluted with 1 × PBS and used directly for NTA assay.

For exosome uptake experiments, exosomes were labeled with the PKH67 fluorescent Cell adaptor kit (Sigma-Aldrich) according to the manufacturer's instructions. That is, 50 microliter exosomes were mixed with 500 μL Diluent C; 2 μL of PKH67 and 500μL Diluent C were mixed and incubated for 4 min. Then 1mL of 1% BSA was added and incubated for 1min, mainly for termination of staining. Labeled exosomes were washed with PBS, collected by ultracentrifugation, and re-suspended in PBS. Exosomes were incubated with HCC cells and analyzed using confocal microscopy at the indicated time points.

### Western blot analysis

Total proteins from cells or exosomes were extracted using RIPA lysis buffer containing protease inhibitor (A32955, Thermo). Protein lysates were then quantified using the BCA kit (#23227, Thermo Fisher Scientific). Proteins were separated by electrophoresis using 10% sodium dodecyl sulfate polyacrylamide (SDS-PAGE) and transferred to PVDF membranes (Millipore). The membranes were blocked with 5% skim milk for 1 h at room temperature and then incubated overnight at 4°C with the following primary antibodies: CD63 (ab217345, 1:1000; Abcam), CD81 (#66866-1-Ig, 1:1000; Proteintech), Calnexin (ab22595, 1:1000; Abcam), GAPDH (ab8245, 1:5000; Abcam). The membranes were then incubated with horseradish peroxidase-conjugated secondary antibodies of the corresponding species for 1 h at room temperature and subjected to electrochemiluminescence development by chemiluminescence instrumentation. Antibodies and reagents are detailed in Supplementary Table 1.

### RNA extraction and quantitative reverse transcription PCR (qRT-PCR)

Total RNA was extracted using the TRIzol Reagent (#15596018, Life Technologies), according to the manufacturer's instructions, then reverse transcribed into cDNA using reverse transcriptase master mix (R312-00, Vazyme). The cDNA was subjected to qRT-PCR, performed on CFX96 qPCR system (Bio-Rad) using the SYBR Green Real-time PCR Master Mix (Q712-02, Vazyme) according to the manufacturer's instructions. The primers used in this study were synthesized by Tsingke Biotechnology Co. Ltd. In this experiment, the relative transcript levels of the target genes were calculated by the 2-△△CT method using GAPDH as the internal reference: △△Ct = △Ct experimental group - △Ct control group, where △Ct = Ct target gene - Ct internal reference. The primer sequences are detailed in Supplementary Table 2.

### Flow cytometry

When the macrophage fusion reached 80-90% after induction, the cells were washed twice with 1×PBS. The cells were then digested with 5 mM EDTA and collected into centrifuge tubes for centrifugation (4°C, 500 × g, 5 min), washed twice with PBS containing 1% FBS, and counted. The cells were then incubated in the dark for 30 min at 4°C with the corresponding antibody. Cells were washed 3 times with PBS. The stained cells were analyzed by flow cytometry (Fortessa, BD Biosciences, USA). Antibodies used for flow cytometry: CD163 (#326505, Biolegend), CD206 (#321109, Biolegend). Data were analyzed by FlowJo software 8.7.1 (Treestar Inc., USA).

### Cell migration assay

The migration and invasion ability of HCC cells was determined by using 24-well Transwell chambers with upper and lower culture chambers separated by polycarbonate membranes with 8 µm pores (BD Biosciences, Franklin Lakes, NJ, USA). 1×10^5^ cells were resuspended in 400 μL of serum-free DMEM medium and inoculated into the upper chamber. The lower chamber was filled with 600 μL of M2 supernatant or exosomes containing 10% FBS, and PBS was used as a control. After incubation for 24 h in a humidified incubator containing 5% CO2 maintained at 37°C, the remaining cells in the upper chamber were removed, and cells migrating to the surface of the lower chamber were fixed with 4% paraformaldehyde and stained with 0.5% crystal violet. At least five random microscopic views (magnification ×100) were taken and the cells were counted. Three independent experiments were performed.

### Wound healing assay

The cells were seeded in a six-well plate. Once the cells became sub-confluent, a 200 μL pipette tip was used to create a scratch wound, after 48 hours of co-culture with the conditioned medium and HCC cells, the width of the scratch was observed and images were obtained under microscope. The results are expressed as a percentage of wound closure.

### Cell proliferation assay

Cell viability was tested with Cell Counting Kit-8 (CCK8) kit (Beyotime) according to the manufacturer's instructions. HCC cells were trypsinized, counted and then plated at a density of 8,000 cells per well in 96-well plates. After that, they were co-cultured with M2 CM. The cells were incubated at 37°C. The attached cells were treated with the CCK8 dilution for 1 hour, and then the absorbance of each well of the plates was measured at 450 nm. For the assay of proliferative capacity of M2 macrophages, 8000 THP-1 cells were spread in 96-well plates and then induced into M2 macrophages, and the cells were treated with a dilution of CCK8 for 1 hr at selected time points, and the absorbance of each well was measured at 450 nm.

### Apoptosis assessment by Annexin V/propidium iodide staining

After induction of THP-1 cells into M2 macrophages. Cells were treated with or without 10μM CPT (camptothecin) for 24 h. The cell supernatant was removed, and then after the cells were washed twice with PBS, 500 μL of staining solution (containing annexin V fluorescein and propidium iodide in the binding buffer ((Annexin V-FITC/PI apoptosis detection kit; Yeasen Biotechnology (Shanghai) Co., Ltd))) was added. After incubation for 15 min at room temperature, pictures were taken using a confocal microscope.

### RNA sequencing and raw data preprocessing

Total RNA from the cells was extracted using Trizol. RNA library construction and sequencing were performed by Berry Genomics Co. Ltd. Then the cDNA library was sequenced on an Illumina Hiseq 2500.

Quantile normalization, log2 conversion, missing values supplement and differential analysis for the matrix data of RNA-Seq dataset were performed using the “limma” package in R/Bioconductor software (version 4.2.1).

### Proteomic analysis

Peptides (300 ng) obtained from M2 exosomes were used for LC-MS analysis. The LC-MS analysis was performed using an EASY nLC1200 FAIMS Orbitrap Exploris 480 mass spectrometer (Thermo Fisher Scientific, USA). The MS system uses Data Independent Acquisition (DIA) for scanning. The raw LC-MS/MS data files were analyzed using Spectronaut (version 17.2.230208.55965) with the spectra searched against the UniProt human database. Search parameters were set as follows: enzyme digestion: Trypsin/P digestion; variable modifications including: oxidation of methionine (M), protein N-terminal acetylation; immobilised modifications including: urea methylation (C); maximum of two missing trypsin cleavage sites allowed; Filtering for the protein and peptide identification was set at a 1% false discovery rate (FDR).

### Animal studies

The animal care and experimental protocols were approved by the Institutional Animal Care and Use Committee (IACUC) of National Center for Protein Sciences (Beijing), Ethical review number: IACUC-20221209-78MT. NOD-SCID mice were purchased from Charles River, Inc (Beijing, Vital River Laboratory Animal Technology). All mice were 5-6 weeks old males. To examine the metastatic ability of the HCC cells, 6-week-old male NOD SCID mice were intravenously injected via the tail vein with 2 × 10^6^ MHCC-97H cells that had been treated with M2 CM. The number of metastatic lungs, the number of clones in the lungs, and small metastatic foci were compared across the four groups.

### Statistical analysis

R version 4.2.1 was used for displaying and computation of publicly available data, RNA-seq and proteomics graphs using the R-packages ggplot2. Other analysis of data was performed using GraphPad Prism 8.0 software. The Student's t-test was used for two-group comparisons, and one-way or two-way ANOVA was used for comparisons among more than two groups. All experiments were performed at least three times, and the data are presented as the mean ± SD. A p-value of less than 0.05 indicates statistical significance.

## Results

### MNDA abundance positively correlates with poor prognosis, inflammation and metastasis in HCC patients

**Figure 1:**
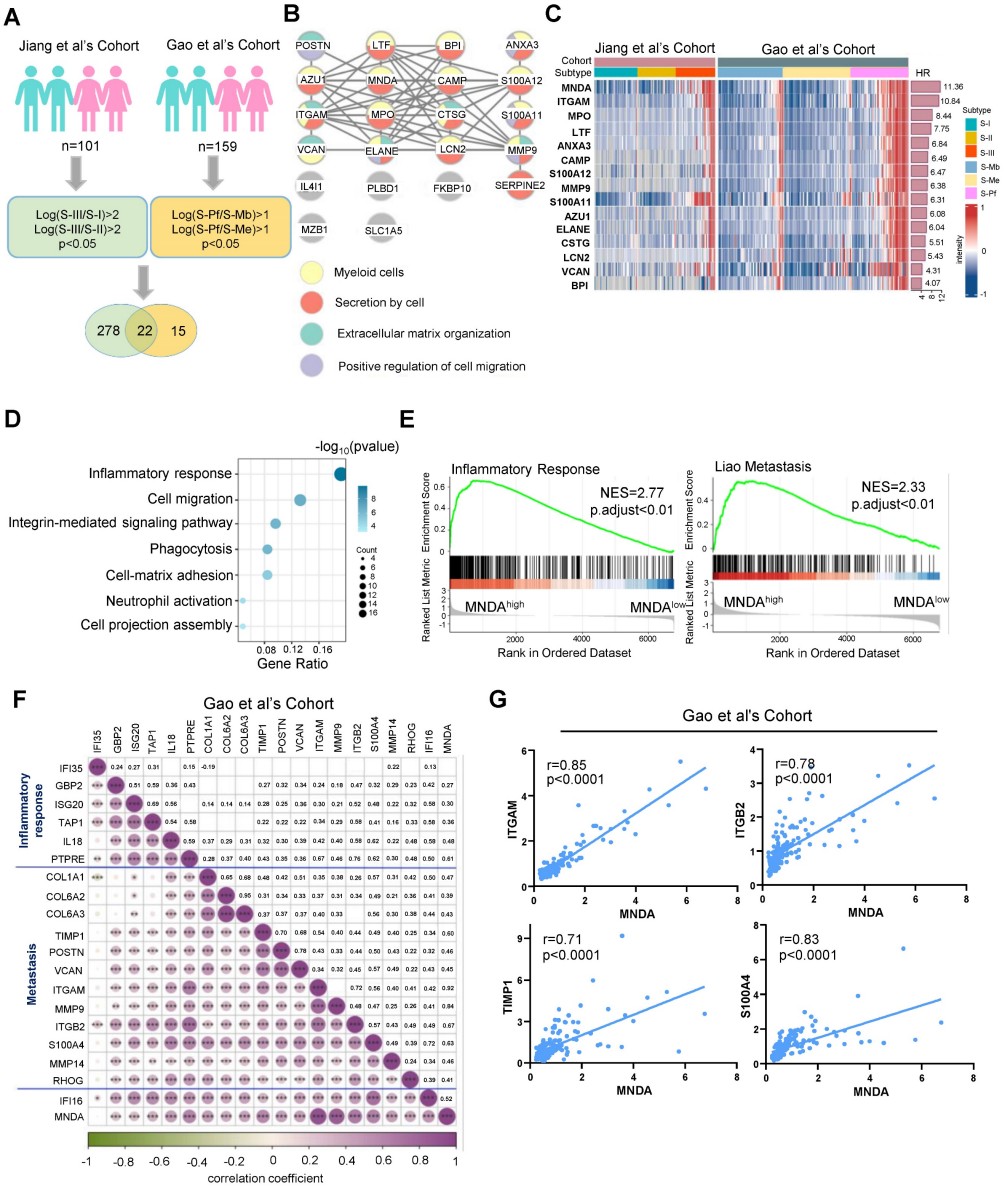
** MNDA abundance positively correlates with poor prognosis, inflammation and metastasis in HCC patients.** (A) Workflow for selecting potential prognostic proteins for S-III/S-Pf HCC. Green represents Jiang et al's Cohort while yellow represents Gao et al's Cohort. The protein filtering conditions are depicted within the rounded rectangle. (B) 22 proteins in the string database for modular analysis. Yellow circles represent myeloid cells; red circles represent secretion by cells; green circles represent Extracellular matrix organization; purple circles represent Positive regulation of cell migration. (C) The heatmap according to HR ordering of significantly differentially expressed 15 proteins (FDR q value < 0.05, t test). HR, hazard ratio (an HR of greater than 1 indicates that the observed variable increases the risk of death). (D) Functional enrichment analysis using the differential proteins between MNDA^low^ group and MNDA^high^ group (Jiang et al.'s cohort: p<0.05 & FC>2; Gao et al.'s cohort: p<0.05&FC>1.5; n=194). (E) GSEA of inflammatory response and Liao metastasis pathways in the MNDA^low^ versus MNDA^high^ groups in the Gao et al's Cohort. (F) The heatmap of MNDA associated with inflammatory response and metastasis-related proteins in the Gao et al's Cohort. (G) Pearson correlation was used to analyze the relationship between MNDA and ITGAM, ITGB2, TIMP1 and S100A4 in Gao et al's Cohort.

In our quest to delve deeper into the common malignant attributes of HCC, we embarked on an analysis aimed at identifying proteins that were highly expressed specifically in the S-III 8 and S-Pf 22 subtypes. Our investigation revealed that, in comparison to the other two subtypes, a total of 22 proteins exhibited significant up-regulation in the S-III/S-Pf subtypes (see schematic overview of the study design in Figure 1A). The 22 proteins were subjected to modular analysis using the STRING database (https://cn.string-db.org/). The analysis revealed that 15 proteins were associated with myeloid cells (Figure 1B), indicating that myeloid cell infiltration could stably predict for poor outcomes in HCC patients. Notably, MNDA showed the highest score indicating a significant prognostic risk (Figure 1C).

Recent research found that aberrant expression of MNDA occurred in lung adenocarcinoma and nodal marginal zone lymphoma 21 24. However, the role of MNDA in HCC remained unclear and needed to be elucidated. To explore the function of MNDA in HCC, we divided patients into two groups: the MNDA^high^ group and the MNDA^low^ group, based on the maximum survival difference of MNDA in proteomic cohorts. Subsequently, we performed functional enrichment analysis using the differentially expressed proteins between the two groups in both cohorts (Jiang et al's Cohort: p<0.05&FC>2; Gao et al's Cohort: p<0.05&FC>1.5; n=194). The results revealed that the signaling pathways were primarily enriched in inflammatory response, cell migration, integrin-mediated signaling pathway etc (Figure 1D). GSEA further revealed that inflammatory response and metastasis were enriched in MNDA^high^ groups in Jiang et al's Cohort and Gao et al's Cohort (Figure 1E and Supplementary Figure 1A). Then, we conducted a rigorous correlation analysis to investigate the relationship between MNDA and a selection of identified immune response and migration-related proteins. Our analysis revealed a robust and significant correlation between MNDA and these proteins, exemplified by ITGAM 25, ITGB2 26, TIMP-1 27, S-100A4 28, 29 (Figures 1F and G and Supplementary Figures 1B and C). These findings suggested the potential role of MNDA in shaping the tumor immune microenvironment, particularly in HCC patients with metastasis.

### MNDA augments the prognostic stratification capability of clinical indicators

**Figure 2:**
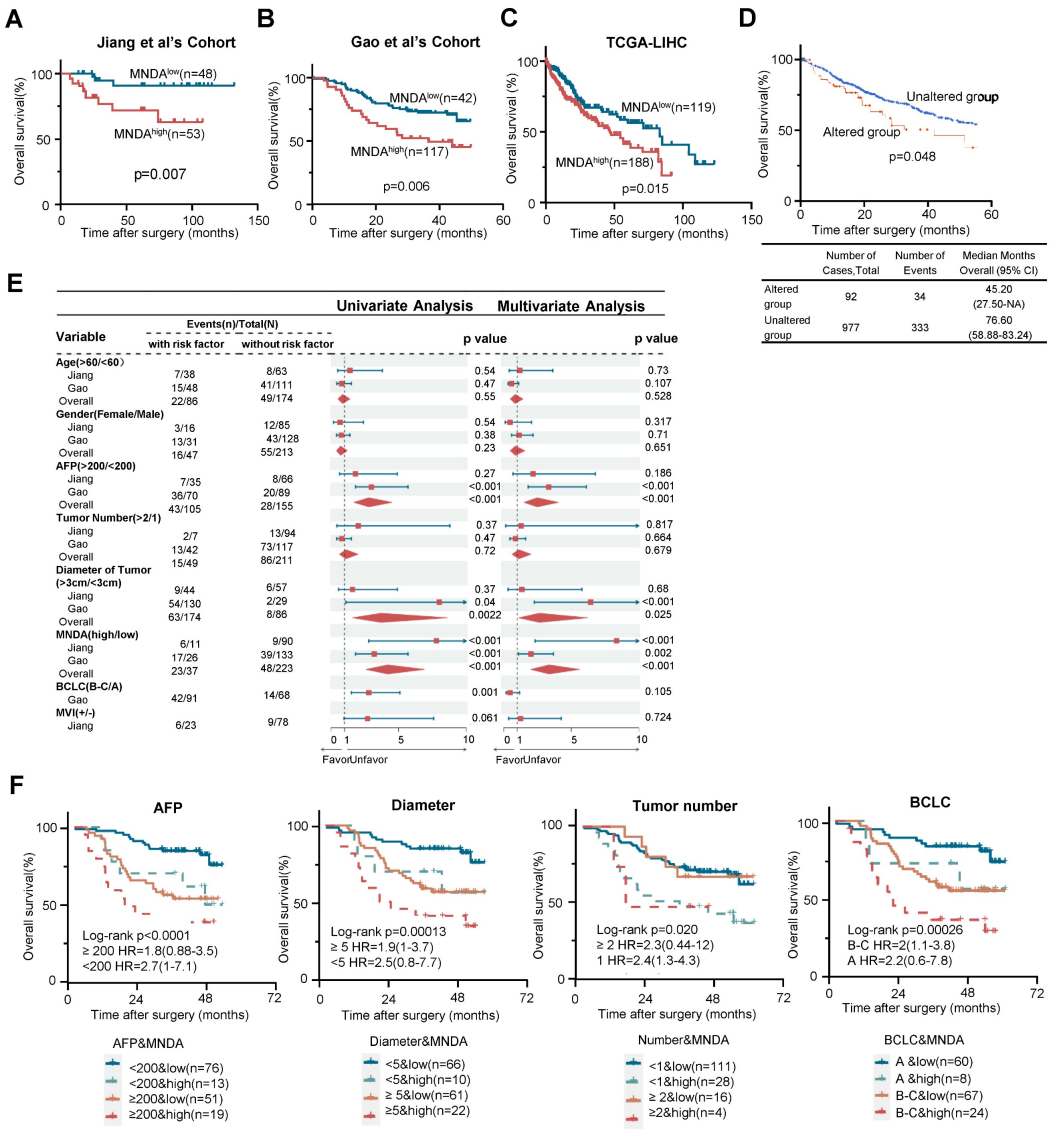
** MNDA augments the prognostic stratification capability of clinical indicators.** (A-C) Kaplan-Meier analysis of the OS probability for HCC patients with different MNDA expression intensities under the optimal cut-off value in Jiang et al's Cohort, Gao et al's Cohort and TCGA-LIHC cohort. (D) Kaplan-Meier analysis of the OS probability for HCC patients with different genomic amplification of MNDA (cBioPortal). (E) Univariate and multivariate variable Cox regression model analysis of MNDA and clinical factors in two proteome cohorts. (F) Kaplan-Meier curves of Gao et al. 's Cohort demonstrating differences in overall survival among patients with AFP<200 ng/mL & MNDA^low^, AFP<200 ng/mL & MNDA^high^, AFP≥200 ng/mL & MNDA^low^, AFP≥200 ng/mL & MNDA^high^; Diameter<5 cm & MNDA^low^, Diameter<5 cm & MNDA^high^, Diameter≥5 cm & MNDA^low^, Diameter≥5 cm & MNDA^high^; Tumor number<1 & MNDA^low^, Tumor number<1 & MNDA^high^, Tumor number≥1 & MNDA^low^, Tumor number≥1 & MNDA^high^; BCLC A& MNDA^low^, BCLC A& MNDA^high^, BCLC B-C& MNDA^low^, BCLC B-C& MNDA^high^.

To investigate the association between MNDA and the prognosis of HCC patients, we first conducted Kaplan-Meier survival analysis using the two proteomic cohorts and The Cancer Genome Atlas (TCGA) dataset. The results revealed that patients with higher MNDA expression, regardless of the protein or mRNA level, exhibited significantly poorer overall survival (OS) (Figures 2A-C). Furthermore, higher levels of MNDA expression were notably correlated with shorter disease-free survival (DFS) in HCC patients, as observed in Jiang et al's Cohort (Supplementary Figure 2A). Consistently, the analysis conducted on https://www.cbioportal.org/ revealed that patients with genomic amplification of MNDA had worse OS and DFS compared to those with unaltered genomic amplification of MNDA (Figure 2D and Supplementary Figure 2C). In the two proteomics cohorts, MNDA exhibited a substantial up-regulation in the S-III/S-Pf subtypes compared with adjacent tissues or other HCC subtypes. This underscored that the distinctive expression patterns of MNDA were closely associated with the degree of malignancy of HCC (Supplementary Figure 2D). Together, these results suggested that the elevated levels of MNDA were commonly found in highly aggressive HCC cases, correlating with a poor prognosis.

Seven factors (i.e., Age, Gender, AFP, Tumor number, Tumor size, BCLC stages, and MVI) were identified as statistically significant factors associated with overall survival (OS) in HCC patients. To further investigate the role of MNDA in clinical diagnosis, we carried out univariate and multivariate analyses. The results indicated that the performance of MNDA as a prognostic marker surpassed most clinical indicators (Figure 2E). Furthermore, MNDA had no significant correlation with current clinical indicators that suggest prognosis (such as AFP, tumor size, MVI, etc.), this data implied that MNDA could serve as an independent prognostic factor (Supplementary Figure 2E). The co-occurrence of high MNDA levels and clinical indicators was associated with a significantly worse overall survival (OS) in HCC patients, as illustrated in Figure 2F and Supplementary Figure 2F. Taken together, these data suggested that MNDA augments the prognostic stratification capability of clinical indicators.

### MNDA is mainly expressed in M2 macrophages

**Figure 3:**
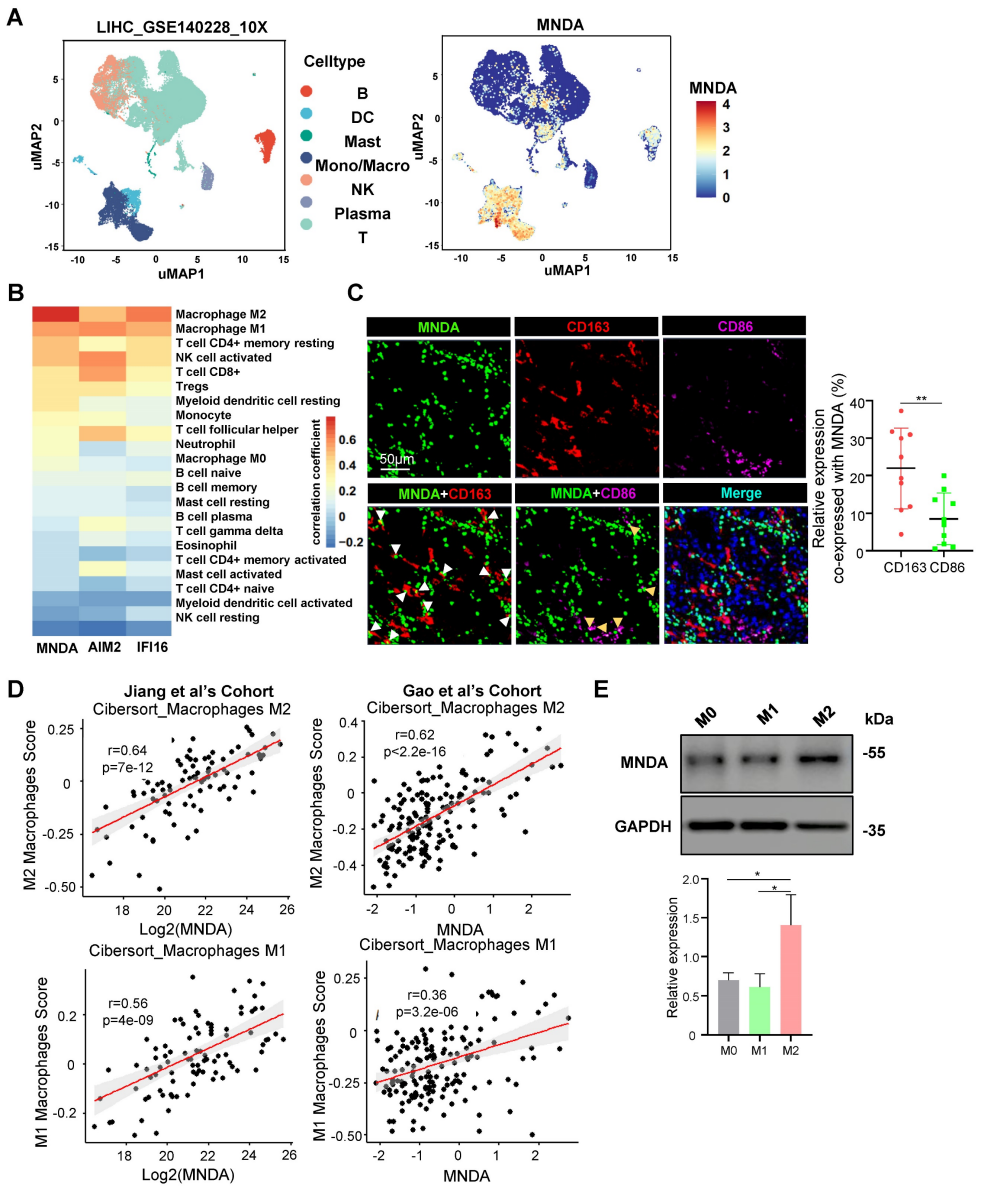
** MNDA is mainly localized in M2 macrophages.** (A) Clustering of cell clusters in the LIHC microenvironment using the uniform manifold approximation and projection (UMAP) method in the GSE140228 database. (B) Analysis of MNDA and its family members in TCGA data using the CIBERSORT-ABS method with 22 immune cell correlations. (C) Confocal microscope image (left) of immunostaining for MNDA (green), CD163 (red), and CD86 (purple), alongside statistical analysis (right) of CD163 or CD86 co-expression with MNDA on tissue sections from 10 patients diagnosed with hepatocellular carcinoma. (D) Spearman correlation was used to analyze the relationship between MNDA and M1/M2 in two proteome cohorts. (E) Western blot analysis and gray scale statistics of MNDA expression in M0/M1/M2 macrophages. Cell experiments were repeated three times independently.

To explore the function of MNDA involved in HCC progression, we firstly examined the expression features of MNDA. MNDA is expressed only in myeloid rather than hepatoma cell lines (Supplementary Figure 3A). Furthermore, we applied a Smart-seq-based scRNA-seq method to study the expression of MNDA in 7 immune cell types which including B cells, dendritic cells (DCs), mast cells, monocytes/macrophages, natural killer (NK) cells, plasma and T cells, and found that MNDA was mainly expressed in monocytes/macrophages (Figure 3A). Moreover, according to the data from the online website (TIMER 2.0), unlike other HIN-200 families, MNDA was found to have the highest correlation with M2 macrophages (Figure 3B). To further validate these findings, we conducted multiplexed immunofluorescent staining, revealing a greater co-localization of MNDA with CD163^+^ M2 macrophages in comparison to CD86^+^ M1 macrophages (Figure 3C). The association between MNDA and M2 expression from proteomic cohorts also showed that MNDA was positively correlated with M2 infiltration rather than M1 infiltration (Figure 3D).

The differentiated THP-1 monocytes are widely used as *in vitro* models of human macrophages 30, and to unveil the expression of MNDA in polarized macrophages, M0, M1 and M2 macrophages were differentiated from THP-1 cells. The upregulation of the pan macrophage marker *CD68* indicated that the M0 macrophage phenotype was successfully differentiated (Supplementary Figure 3B). In induced M1 macrophages, the markers for M1 (*IL6*, *CXCL10*) rose compared to M0 and M2 macrophages, while the markers for M2 (*IL-10*, *CCL22* and *CD206*) rose only in induced M2 macrophages (Supplementary Figures 3C and D). The flow cytometry analysis also showed a higher level of CD206 and CD163 in M2 macrophages (Supplementary Figures 3E and F). These results confirmed the success of the cellular M1 and M2 polarization model establishment we used. In differentiated and induced M0, M1 and M2 macrophages, the expression of MNDA was examined and the results showed that MNDA was significantly highly expressed in M2 macrophages (Figure 3E). Taken together, these results confirmed the high expression of MNDA in M2 macrophages.

M2 macrophages share numerous characteristics with TAMs and exhibit several key features associated with malignant tumors, including angiogenesis, invasiveness, metastasis, regulation of the TME, and therapeutic resistance 31. Indeed, we found M2 macrophage score had a higher value in S-III subtype, which was associated with the poorest outcome and featured immune dysregulation and tumor metastasis (Supplementary Figure 3G). Consistently, GSEA analysis further revealed the enrichment of the inflammatory response and metastasis pathways in the M2^high^ group by analyzing two independent proteomics datasets (Supplementary Figure 3H).

### MNDA enhances M2 macrophages polarization

**Figure 4:**
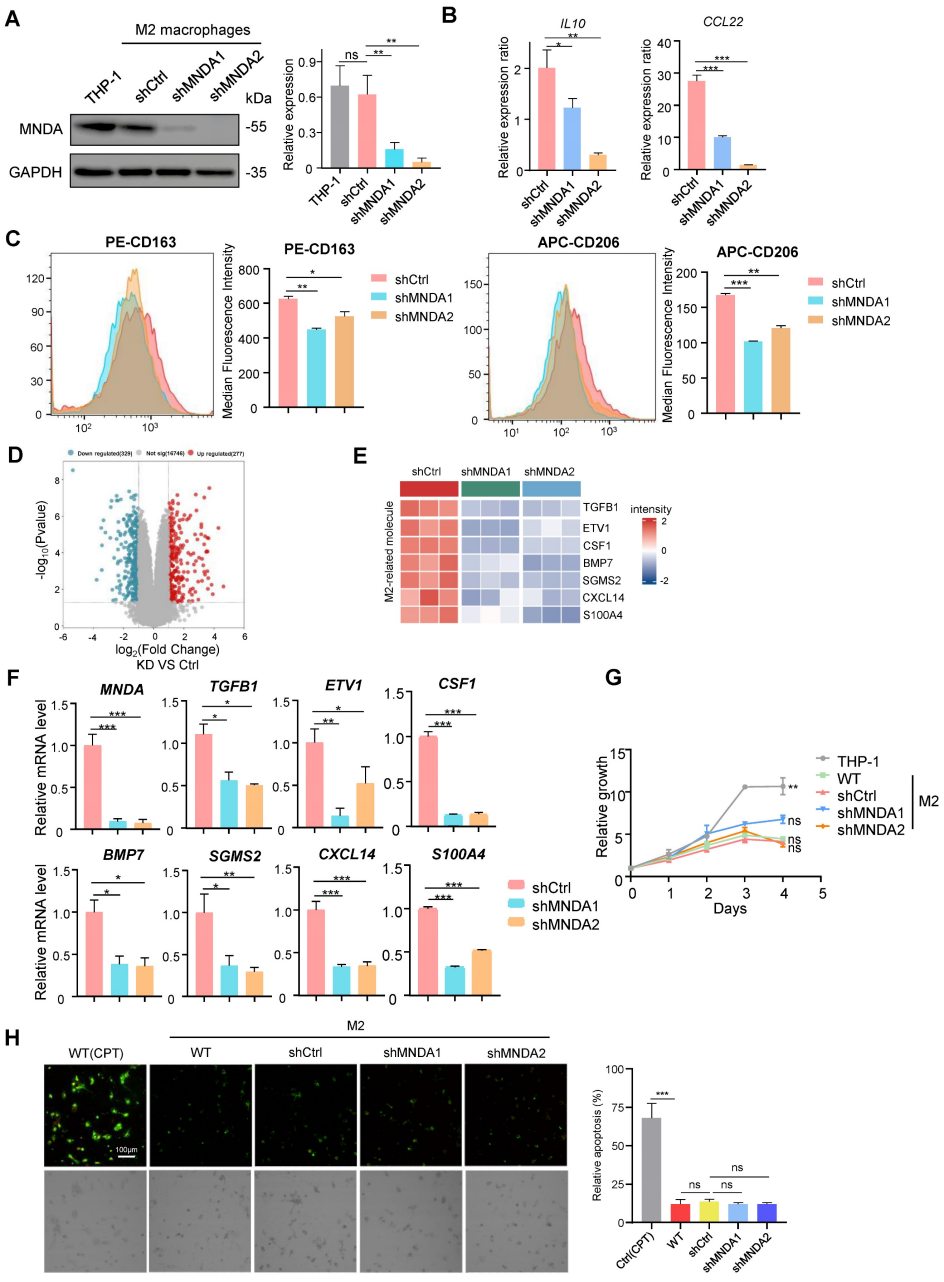
** MNDA enhances M2 macrophages polarization.** (A) Western blotting assay of MNDA expression levels in M2 macrophages induced from THP-1 cells after MNDA silencing. (B) The expression ratio of *IL10* and *CCl22* in M2 macrophages (shCtrl, shMNDA1, and shMNDA2) relative to M0 macrophages was assessed using qPCR analysis. (C) The expression ratio of CD163 and CD206 in M2 macrophages (shCtrl, shMNDA1, and shMNDA2) relative to M0 macrophages was detected by Flow cytometry analysis. (D) A volcano plot of the differential genes in MNDA knockdown and control M2 macrophages in the RNA-Seq data. Blue dots represent the downregulated genes (p < 0.05; logFC<-1.5), red dots represent the upregulated genes (p < 0.05; logFC>-1.5), and gray dots indicate genes with no significant difference. n = 3 (E) A heatmap of the expression of enhancers of protumor macrophage polarization in M2 macrophages with and without MNDA silencing in the RNA-Seq data. (F) qRT-PCR analysis of MNDA, TGFB1, ETV1, CSF1, BMP7, SGMS2, CXCL14 and S100A4 in shCtrl, shMNDA1 and shMNDA2 M2 macrophages. (G) Cell proliferation assay of THP-1 and M2 macrophages induced from shCtrl, shMNDA1 and shMNDA2 THP-1 cells. (H) Fluorescence photographic images (left) and statistic results (right) of apoptosis in Ctrl (CPT), WT, shCtrl, shMNDA1 and shMNDA2 M2 macrophages. Ctrl (CPT): positive control, THP-1 cells were induced into M2 macrophages, followed by the addition of 10µM CPT (Camptothecin). Cell experiments were repeated three times independently. (*P<0.05, **P<0.01, ***P<0.001).

To clarify the role of MNDA in M2 macrophages, we specifically knocked down MNDA in THP-1 cells using lentivirus-mediated gene transfer and established two MNDA stable knockdown THP-1 cell lines (shMNDA1 and shMNDA2) and its control cell (shCtrl). The shMNDA and its control THP-1 cells were then induced for M2 polarization, western blot analysis demonstrated a significant reduction in MNDA expression in both shMNDA1 and shMNDA2 groups (Figure 4A). Subsequently, we conducted qRT-PCR analysis, which revealed a decrease in the expression ratio of M2 macrophage markers (*IL-10*, *CCL22*) in M2/M0 following MNDA knockdown (Figure 4B). Additionally, flow cytometry analysis showed a strong correlation between MNDA deficiency and reduced expression rates of CD163 and CD206 in M2/M0 (Figure 4C), suggesting that MNDA might enhance the induction rates of M2 macrophages. Furthermore, we performed RNA-seq analysis to delve deeper into the functional role of MNDA in M2 macrophages. The transcriptome data showed that 606 genes (329 downregulated and 277 upregulated) were differentially expressed with statistical significance (Figure 4D and Supplementary Table 3). Heatmap showed the significantly downregulation of *TGFB1* 32, *ETV1* 33, *CSF1* 34, *BMP7* 35, *SGMS2* 36, *CXCL14* 37 and *S100A4* 38 which have been reported to enhance protumor macrophage polarization via various pathways. (Figure 4E). We validated our transcriptomics data by qRT-PCR (Figure 4F). Overall, these findings suggested that MNDA might play a regulatory role in the expression of M2-related molecules and contribute to the enhancement of M2 macrophage polarization. At the same time, we further assessed the MNDA's potential role in proliferation and apoptosis using CCK8 assay and Annexin V/Propidium Iodide Staining, respectively, and the results showed that MNDA knockdown did not affect M2 macrophages proliferation and apoptosis (Figure 4G-H).

### MNDA drives the secretion of pro-metastatic proteins by M2 macrophages

**Figure 5:**
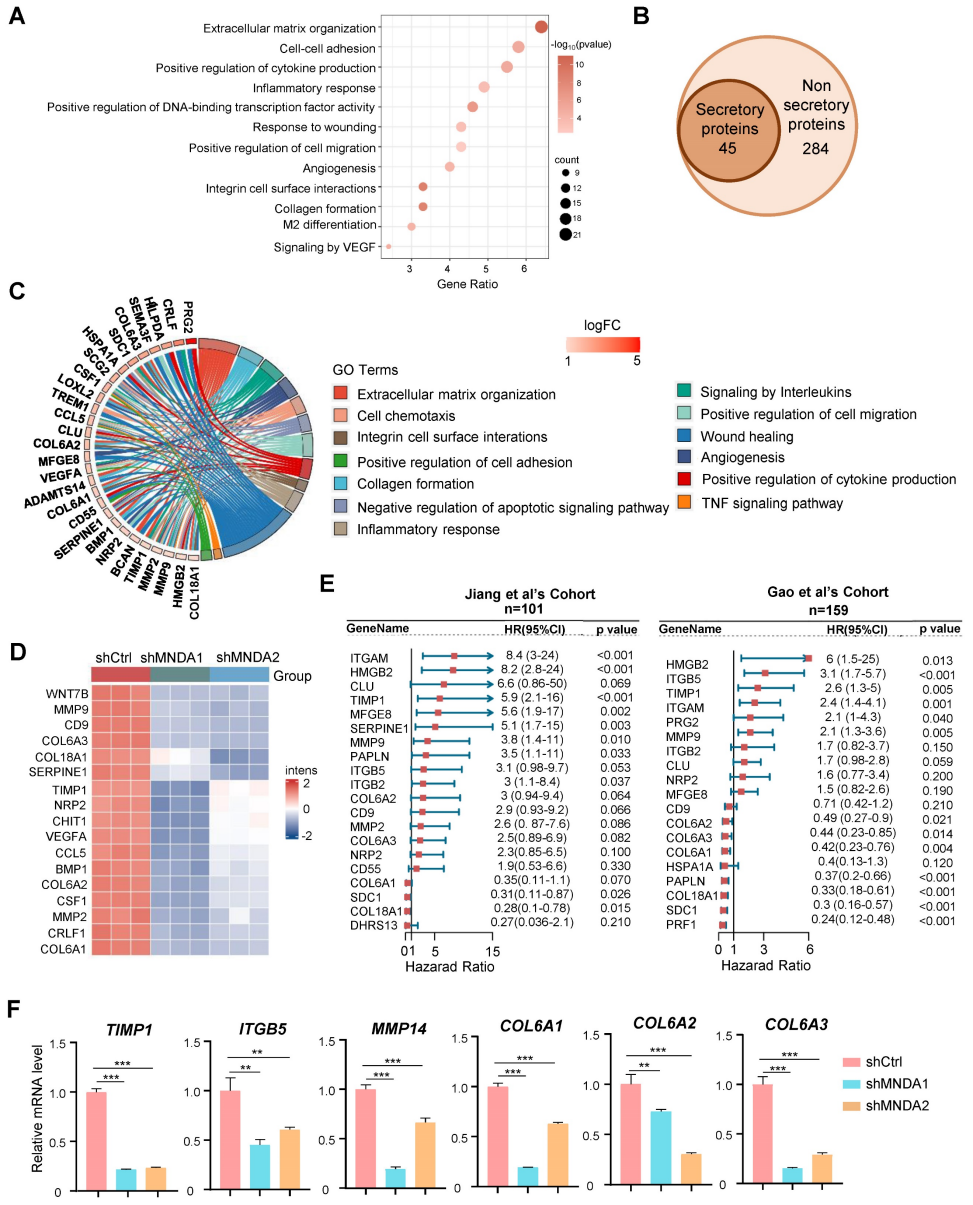
** MNDA drives the secretion of pro-metastatic proteins by M2 macrophages.** (A) Bubble plot of pathway enrichment analysis of differentially expressed genes in M2 macrophages with and without MNDA silencing identified by RNA-Seq. (FDR<0.05; Log (Ctrl/shMNDA)>1). (B) Venn diagram showed 45 secretion-related genes in the 329 down-regulated genes among shMNDA vs shCtrl M2 macrophages. (C) Pathway chord plot of enrichment analysis of secreted proteins in M2 macrophages with and without MNDA silencing identified by RNA-Seq. (D) A heat map of the expression of metastasis-associated genes in shCtrl, shMNDA1 and shMNDA2 M2 macrophages identified by RNA-Seq. (E) Forest plot of survival analysis of secreted proteins in two proteome cohorts. (F) qRT-PCR analysis of *TIMP1*,* ITGB5*, *MMP14*, *COL6A1*, *COL6A2* and *COL6A3* in shCtrl, shMNDA1 and shMNDA2 M2 macrophages. Cell experiments were repeated three times independently. (*P<0.05, **P<0.01, ***P<0.001).

We next proceeded to unveil the modulators of MNDA in M2 macrophages using the transcriptome data. KEGG analysis was performed for genes significantly down-regulated after knockdown of MNDA in M2 macrophages, and the results showed that signaling pathways were mainly enriched in extracellular matrix organization, cell-cell adhesion, cytokine production, inflammatory response, etc (Figure 5A). Strikingly, among the 329 downregulated genes, 45 genes were identified as secretory proteins, which were closely associated with extracellular matrix organization, wound healing, and cell migration (Figures 5B and C). Heatmap showed 17 proteins that associated with tumor metastasis in the RNA-Seq data, were significantly down-regulated with the knockdown of MNDA (Figure 5D). Consistently, the proteome data indicated that high expression of most of the proteins was associated with a poor prognosis for HCC patients (Figure 5E). qRT-PCR confirmed that MNDA positively affected the expression of some pro-metastasis genes (*TIMP1, ITGB5, MMP14, COL6A1, COL6A2* and* COL6A3*) in M2 macrophages (Figure 5F). These data demonstrated that MNDA positively regulated the secretion of pro-metastasis proteins by M2 macrophages. Therefore, we hypothesized that MNDA might promote invasion and migration of HCC cells by regulating the expression of pro-metastatic secretory proteins from M2 macrophages.

### MNDA promotes HCC cells invasion and migration via serum-derived from M2

**Figure 6:**
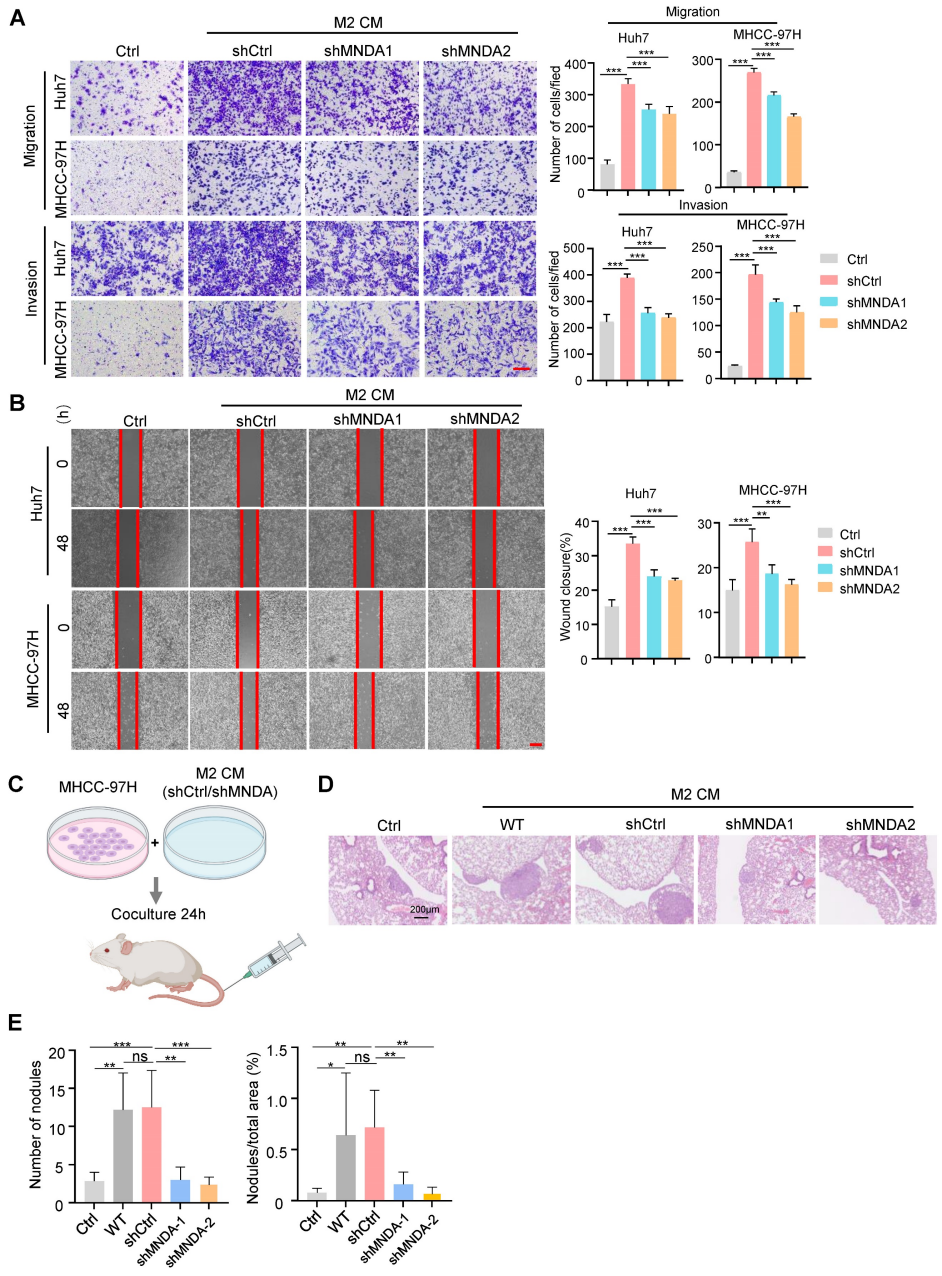
** MNDA promotes HCC cells invasion and migration via serum-derived from M2.** (A) Transwell chamber assay was used to test cell migration and invasion of Huh7 and MHCC-97H cells co-cultured with Ctrl and CM from shCtrl, shMNDA1 and shMNDA2 M2 macrophages. Scale bar, 200μm. (B) Effects of treatment with Ctrl and CM from shCtrl, shMNDA1 and shMNDA2 M2 macrophages on migration of Huh7 and MHCC-97H cells were assessed by wound-healing assay. Images were taken at 0 and 48 h after scratching (left panel represents representative images; right panel shows percentage of migrated distance quantitatively). (C) MHCC-97H cells co-cultured with Ctrl and CM from WT, shCtrl, shMNDA1 and shMNDA2 M2 macrophages were injected into NOD-SCID mice through the tail vein, and the number of pulmonary metastatic nodules was calculated 8 weeks later (n=6 each group). (D) Hematoxylin and eosin (H&E) staining representative images of lung metastasis sites in the tissue samples. (E) The number of lung metastatic nodules and the percent of nodules/total areas in the tissue samples. Scale bar, 200 μm. Cell experiments were repeated three times independently. (*P<0.05, **P<0.01, ***P<0.001; ns, no significance). Ctrl, DMEM medium containing 10% serum.

To confirm the assumption, next, we collected the conditioned medium (CM) from the control and MNDA knockdown M2 macrophages. The results of transwell assays showed that the incubation with CM from the shCtrl and WT M2 macrophages increased the migration abilities of tumor cells (Huh7, MHCC-97H), and the capability of shCtrl and WT M2 macrophages derived supernatants to promote HCC cell metastasis was comparable (Supplementary Figure 4A), whereas the promotion of the CM from the MNDA knockdown cells on tumor cell migration and invasion was weakened (Figure 6A and Supplementary Figure 4B). Similarly, in scratch test, CM derived from M2 macrophage-treated HCC cells showed increased mobility, and the culture with shMNDA M2 CM led to a significant reduction in the HCC cell mobility potential compared with M2 CM-treated cells (Figure 6B and Supplementary Figure 4B). We subsequently co-cultured with the HCC cells with CM from M2 macrophages, and the proliferation displayed no clear alterations (Supplementary Figure 4D). To further elucidate the effect of MNDA on *in vivo* metastasis ability of HCC cells, we intravenously injected MHCC-97H cells that had been pre-incubated with M2 CM into NOD-SCID mice (Figure 6C). Subsequently, we monitored the development of the lung metastasis in these mice. 8 weeks later, histological analysis of the lung tissues revealed a significant increase in the incidence of lung metastases in mice inoculated with MHCC-97H cells treated with M2 CM. However, the promotion of CM from MNDA downregulated M2 macrophages on tumor cell lung metastases was weakened (Figures 6D and E). Notably, there was no significant difference in body weight between the different groups of mice (Supplementary Figure 4E). These findings indicated that MNDA had the potential to enhance HCC cell metastasis both* in vivo* and* in vitro* via regulating the expression of secretory proteins from M2 macrophages.

### MNDA promotes HCC cells migration via exosome-derived from M2

Emerging evidence suggests the central role of exosomes in intercellular communication in tumor metastasis 39. To further study the effect of exosomes on the biological functions of MNDA on HCC cells *in vitro*, we added a specific exosome secretion inhibitor GW4869 to the M2 macrophage culture medium. Transwell assays revealed that treatment with GW4869 decreased the promotion of M2 CM on the migration abilities of Huh7, PLC and MHCC-97H HCC cells (Supplementary Figure 5A). These results indicated that M2 exosomes might be accountable for the effects exerted by M2 macrophages on HCC cells.

**Figure 7:**
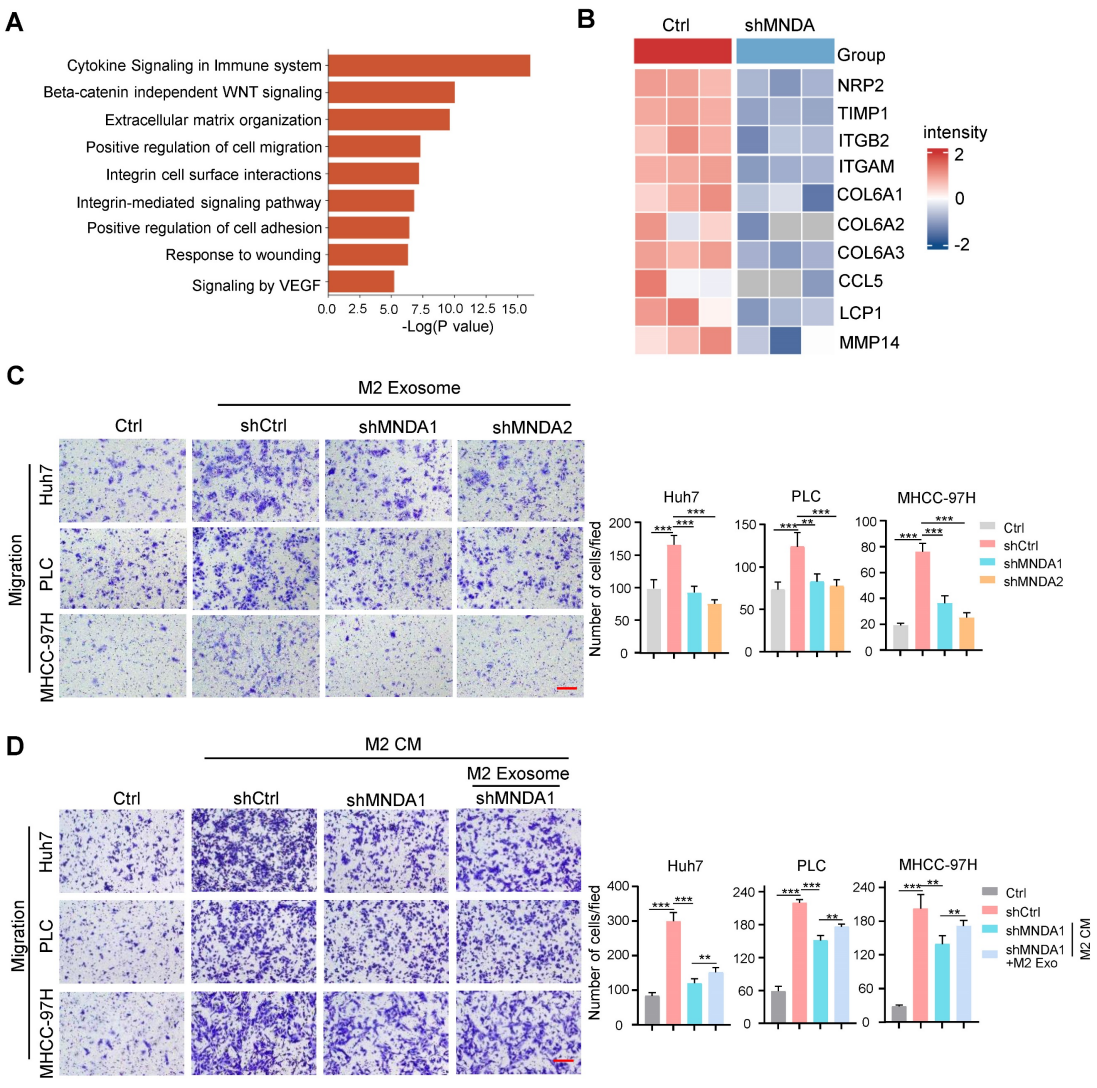
** MNDA promotes HCC cells migration via exosome-derived from M2.** (A) GO analysis showed the biological pathways enriched by differentially expressed proteins in (p<0.05, LogFC<0) M2 macrophages with and without MNDA silencing. (B) Heatmap showed the metastasis-related protein expression in Ctrl and shMNDA M2 exosomes in mass spectrometry data. (C) Transwell chamber assay was used to test cell migration of Huh7, PLC and MHCC-97H cells co-cultured with M2 exosomes derived from shCtrl, shMNDA1 and shMNDA2. Scale bar, 200 μm. (D) Effects of treatment with CM from Ctrl, M2 shCtrl, M2 shMNDA1 and M2 shMNDA1added with M2 macrophage-derived exosomes on the migration ability of Huh7, PLC and MHCC-97H cells, as assessed by a transwell assay. Scale bar, 200 μm. Cell experiments were repeated three times independently. (*P<0.05, **P<0.01, ***p<0.001). Ctrl, HCC cells co-cultured with DMEM medium containing 10% serum.

Subsequently, we extracted exosomes from M2 macrophage supernatants by ultracentrifugation, and western blot results showed that protein levels of the exosome markers CD63 and CD81 were significantly increased in the extracted exosomes compared with cell lysis, whereas the exosome-negative marker calnexin was not detectable in the extracted exosomes (Supplementary Figure 5B). Transmission electron microscopy results showed that the shape of the extracted exosomes had a typical two-layer membrane structure, and nanoparticle tracking analysis (NTA) results indicated that the average diameter of exosomes was 132 nm (Supplementary Figures 5C and D). The above results confirmed the success of exosome extraction. We next detected whether these exosomes could be internalized by HCC cells. Exosomes were labeled with PKH67, a green fluorescent carbocyanine dye, which was followed by the treatment of HCC cells with these labeled exosomes. We used a fluorescence microscope to confirm that PLC cells and Huh7 cells could take up exosomes derived from M2 macrophage with a robust, time-dependent method (Supplementary Figure 5E). To gain insight into the molecular mechanism of MNDA, we studied the regulation of MNDA on the M2 derived exosome. The exosomes from shCtrl and shMNDA of M2 macrophages were isolated and the protein expression profiles in M2 exosomes were analyzed by using mass spectrometry assay (Supplementary Table 4). The results showed dramatic differential expression of proteins between the two sets, and the differential proteins were closely related to cytokine signaling, β-catenin independent WNT signaling, extracellular matrix organization and cell migration (Figure 7A). In particular, a panel of proteins, including NRP2, TIMP1, ITGB2, ITGAM, COL6A1, COL6A2, COL6A3, CCL5 and LCP1, were significantly down-regulated in M2 shMNDA exosomes (Figure 7B). As shown in Supplementary Table 5, the higher expression of these proteins predicted poorer OS and DFS.

We explored whether MNDA has a critical role in promoting the migratory potential of HCC cells via M2-derived exosomes. The exosomes derived from shCtrl, shMNDA1 and shMNDA2 of M2 macrophages were collected and used in the function experiments *in vitro*. Transwell assays showed that the exosomes derived from MNDA knock down M2 macrophages attenuated the migration of Huh7 cells and PLC cells (Figure 7C). Importantly, the addition of M2 exosomes partially restored the weakened promotion of cell migration resulting from the downregulation of MNDA in M2 macrophages (Figure 7D). The aforementioned results suggested that MNDA promoted the migration of HCC cells through exosomes derived from M2 macrophages.

## Discussion

Liver cancer is the third-highest cause of cancer-related deaths worldwide, and HCC is the predominant form of liver cancer 1. Despite treatments with sorafenib, regorafenib and immune checkpoint blockades (ICBs) benefits for other cancer indications, the response rates in HCC were unsatisfactory 40. The complexity and heterogeneity of TME is one of the important reasons. Nevertheless, HCC is an inflammation-driven disease with underlying chronic liver inflammation and cirrhosis, a quarter of HCC cases express markers of inflammatory response 41. It has been reported that the immunosuppressive features of tumor lesions participate not only as one of the major players inducing cancer progression, but also a major challenge for effective immunotherapy resistance 42. Consequently, there is an urgent need to characterize the molecular characteristics of tumor immunosuppressive environment to explore biomarkers and targets of tumor-infiltrating immune cells in HCC.

In this study, based on HCC proteomic data from two independent centers, we found that the most malignant HCC (S-III/S-Pf) were characterized by immune disturbances dominated by myeloid cell infiltration. Particularly, TAMs represent the most abundant immune population in the myeloid cells and are widely considered to be M2-like macrophages that promote tumor progression and suppress local immunity 41. To further explore the potential biomarkers and molecular mechanisms of high M2 infiltration and the crosstalk of M2 with HCC, MNDA attracted our attention.

MNDA is a nuclear factor initially identified in normal and transformed cells of the human myelo-monocytic lineage (granulocytes, monocytes, and macrophages) and its use as a marker of myeloid cell differentiation has been proposed 42. Increasing evidence shows that MNDA expression is associated with clinicopathological features in patients with tumors, yet the function of MNDA in HCC has not been reported. In this study, we found that MNDA as one of the representative proteins of myeloid cells was elevated significantly in the most malignant S-III/S-Pf subtype HCC and positively correlated with M2 infiltration. To explore the association between MNDA and poor prognosis in HCC patients, multiple omics data had been used. We observed patients with high MNDA expression had shorter OS than those with low MNDA expression in both DNA, mRNA and protein levels. In addition, MNDA as an independent prognostic indicator was superior to most clinical markers (AFP level, tumor diameter, number of tumors, BCLC stage, and presence of MVI). To further evaluate the potential diagnostic value of MNDA in HCC, we found that combining MNDA with clinical indicators could further predict the adverse prognosis of HCC.

Interestingly, we found that mainly M2 macrophages-expressed MNDA could promote M2 macrophages polarization and HCC cells metastasis. These data suggested that a higher expression of MNDA might serve as a therapeutic target for the TME of HCC. To decipher the mechanisms underlying how MNDA modulates TAMs with protumor functions, our RNA-seq analysis revealed that reducing macrophagic MNDA could reverse the typical M2-like gene expression signature. Meanwhile, the decreased expression of MNDA also reduced the factors associated with alternative polarization toward a protumor phenotype, such as *CSF1*, *S100A4*. Therefore, it is logical that any intrinsic or extrinsic factors that can induce MNDA upregulation may induce TAM protumor polarization and the poor prognosis of tumor. On the other hand, it has been well-illustrated that TAMs release a wide range of chemokines and cytokines, which potentiate tumor invasion and metastasis 43. In this study, MNDA regulated the expression of 45 secretory genes in M2 macrophage, which were associated with extracellular matrix organization and cell migration. A large number of secretory factors were significantly up-regulated in the proteome cohorts. Furthermore, we demonstrated that MNDA promoted the invasion and migration of HCC cells via serum-derived M2 macrophages.

Exosomes are usually constituted by small vesicles that are released from a variety of cell types 44. Functionally, exosomes can transfer biomolecules (such as proteins, RNAs and DNAs), which have intriguing and elaborate roles in TMEs 45. The dysfunction of exosomes has been widely investigated in HCC development 46. Here, we show the evidence of M2 macrophage-derived exosomes in malignant progression of HCC. For example, macrophages-derived exosomes transmit miR-92a-2-5p to liver cancer cells to increase the invasion capacity of liver cancer 47. In addition, Xu et al. found that macrophage-mediated regulation of the metabolic reprogramming in HCC cells via the exosomal delivery of oncogenic lncMMPA 48. In this study, we firstly revealed that the level of some proteins (such as NRP2, TIMP1, ITGB2, ITGAM) associated with tumor metastasis promotion was decreased in the exosomes derived from MNDA knockdown M2 macrophages, which could be taken up by HCC cells. These findings provided the groundwork for macrophage exosomes research toward a further understanding of their clinical and pathological importance.

## Conclusions

**Figure 8:**
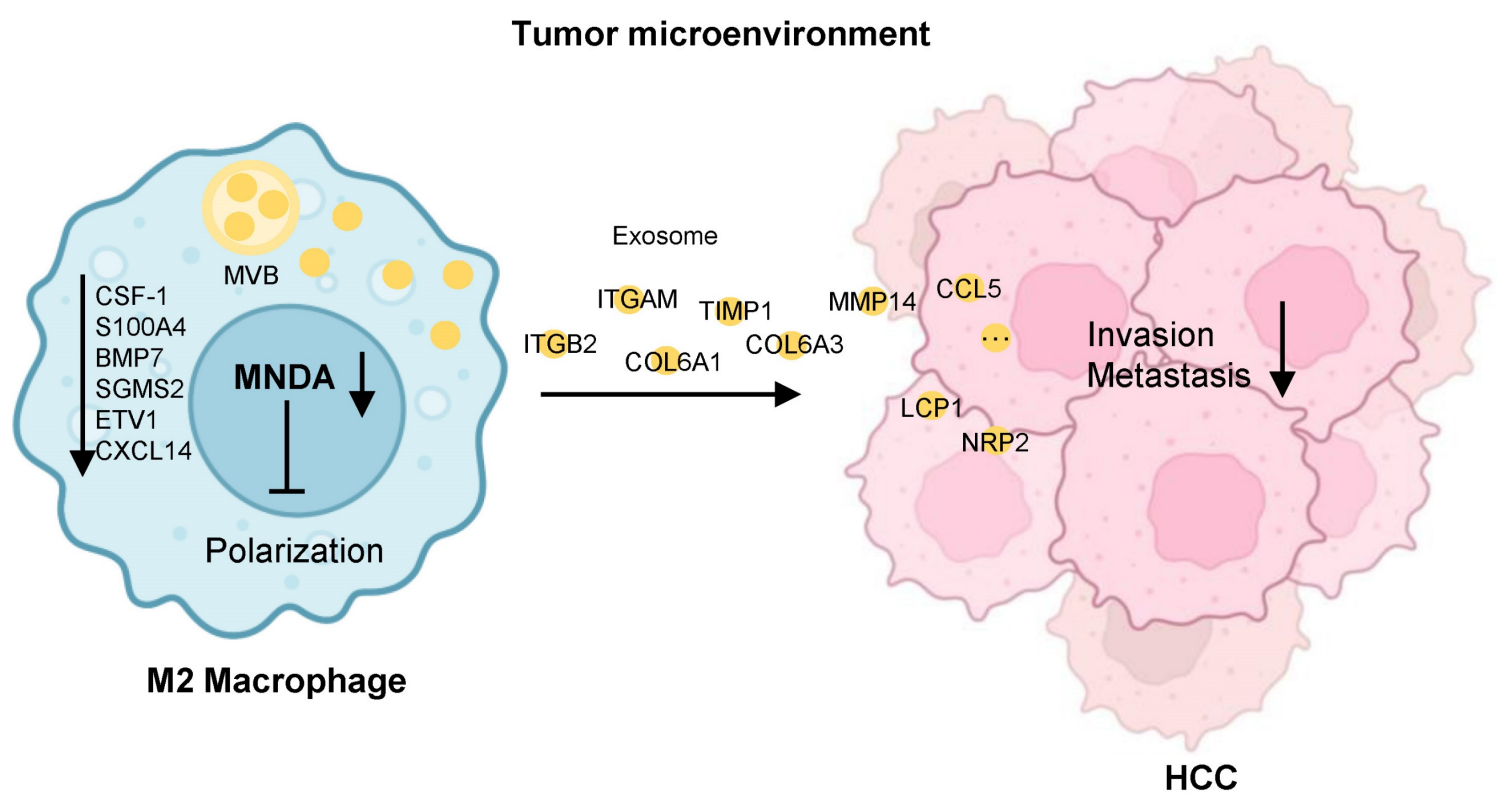
** Schematic diagram summarising the role of MNDA in HCC lung metastasis.** MNDA was primarily expressed in tumor M2 macrophages and contributed to the enhancement of M2 macrophage polarization by upregulating the expression of the enhancers of M2 polarization. MNDA knockdown inhibited the secretion of M2 macrophage-derived pro-metastasis proteins via the exosome pathway to suppress HCC metastasis. MVB: multivesicular body.

In conclusion, our study delineated that MNDA enhanced M2 macrophage polarization by upregulating the enhancers of protumor macrophage polarization and promoted HCC cell metastasis by delivering the pro-metastasis proteins via M2 derived exosomes (Figure 8). Moreover, MNDA could serve as a stratification biomarker and potential therapeutic target for HCC with higher M2 infiltration and poorer prognosis, in attempts to improve the survival rates of these patients.

## Supplementary Material

Supplementary figures and tables.

## Abbreviations

MNDA: Myeloid cell nuclear differentiation antigen
HCC: Hepatocellular carcinoma
TAM: Tumor-associated macrophages
TIME: Tumor immune microenvironment
HR: Hazard ratios
GSEA: Gene set enrichment analysis
ES: Enrichment score
TCGA: The Cancer Genome Atlas
OS: Overall survival
DFS: Disease-free survival
NTA: Nanoparticle tracking analysis
CM: conditioned media
S-Mb: metabolism subgroup
S-Me: Microenvironment dysregulated subgroup
S-Pf: Proliferation subgroup
IL4: Interleukin-4
IFN-γ: Interferon-γ
PMA: Phorbol 12-myristate 13-acetate
