# Supplementary material for: Myeloid cell-expressed MNDA enhances M2 polarization to facilitate the metastasis of hepatocellular carcinoma
Source: Int J Biol Sci. 2024 May 5;20(8):2814–32. doi: 10.7150/ijbs.91877 (PMC11186364; doi:10.7150/ijbs.91877)
Supplement: Supplementary file 1 — Supplementary figures and tables. [file ijbsv20p2814s1.zip › Supplementary figure legends.pdf]

Supplementary Fig. 1

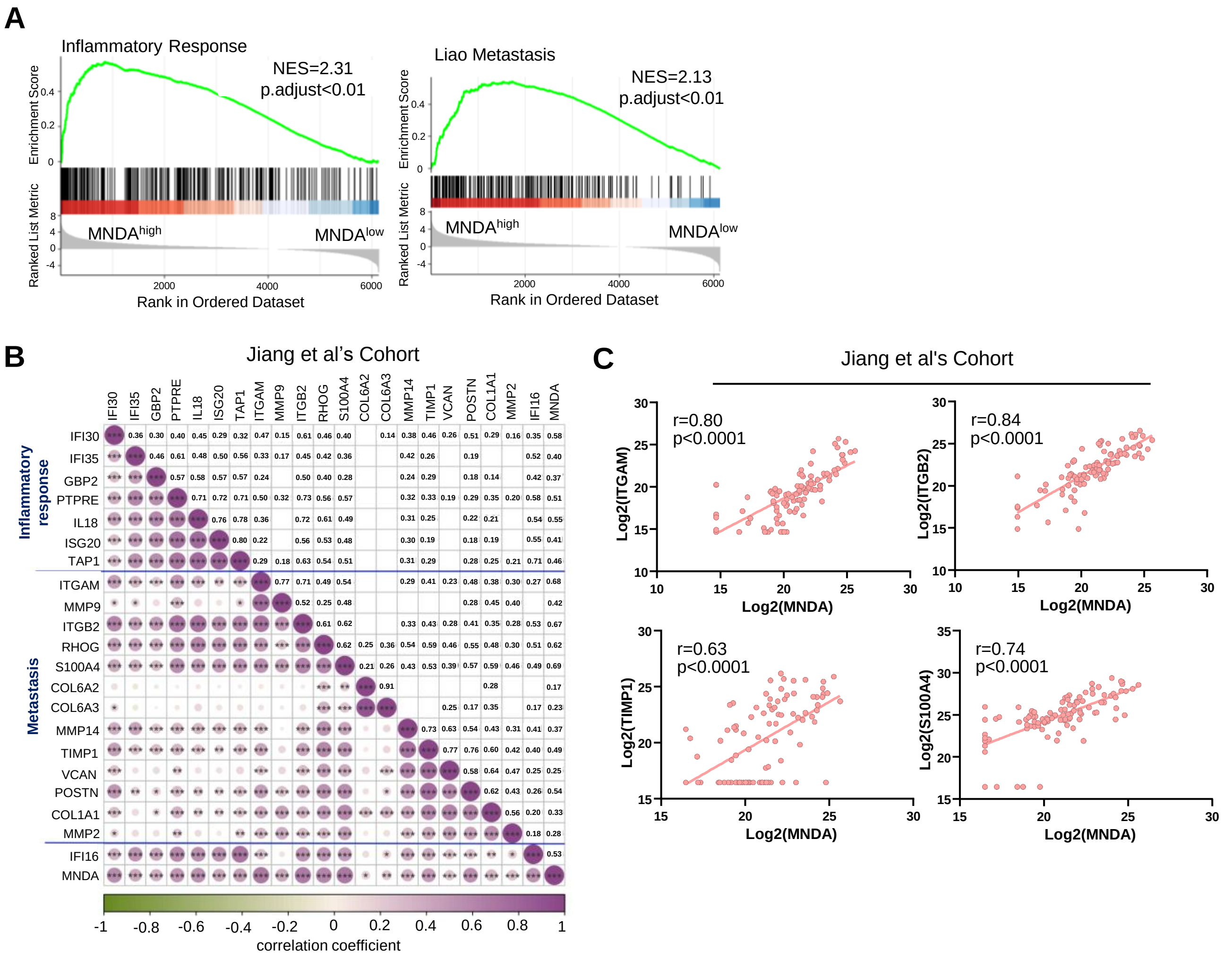

Supplementary Fig. 1 MNDA abundance positively correlates with poor prognosis, inflammation and metastasis in HCC patients. (A) Gene set enrichment analysis (GSEA) of inflammatory response and Liao metastasis pathways in the $MNDA^{low}$ versus $MNDA^{high}$ groups in the Jiang et al's Cohort. (B) The heatmap of MNDA associated with inflammatory response and metastasis-related proteins in the Jiang et al's Cohort. (C) Pearson correlation was used to analyze the relationship between MNDA and ITGAM, ITGB2, TIMP1 and S100A4 in the Jiang et al's Cohort.

Supplementary Fig. 2

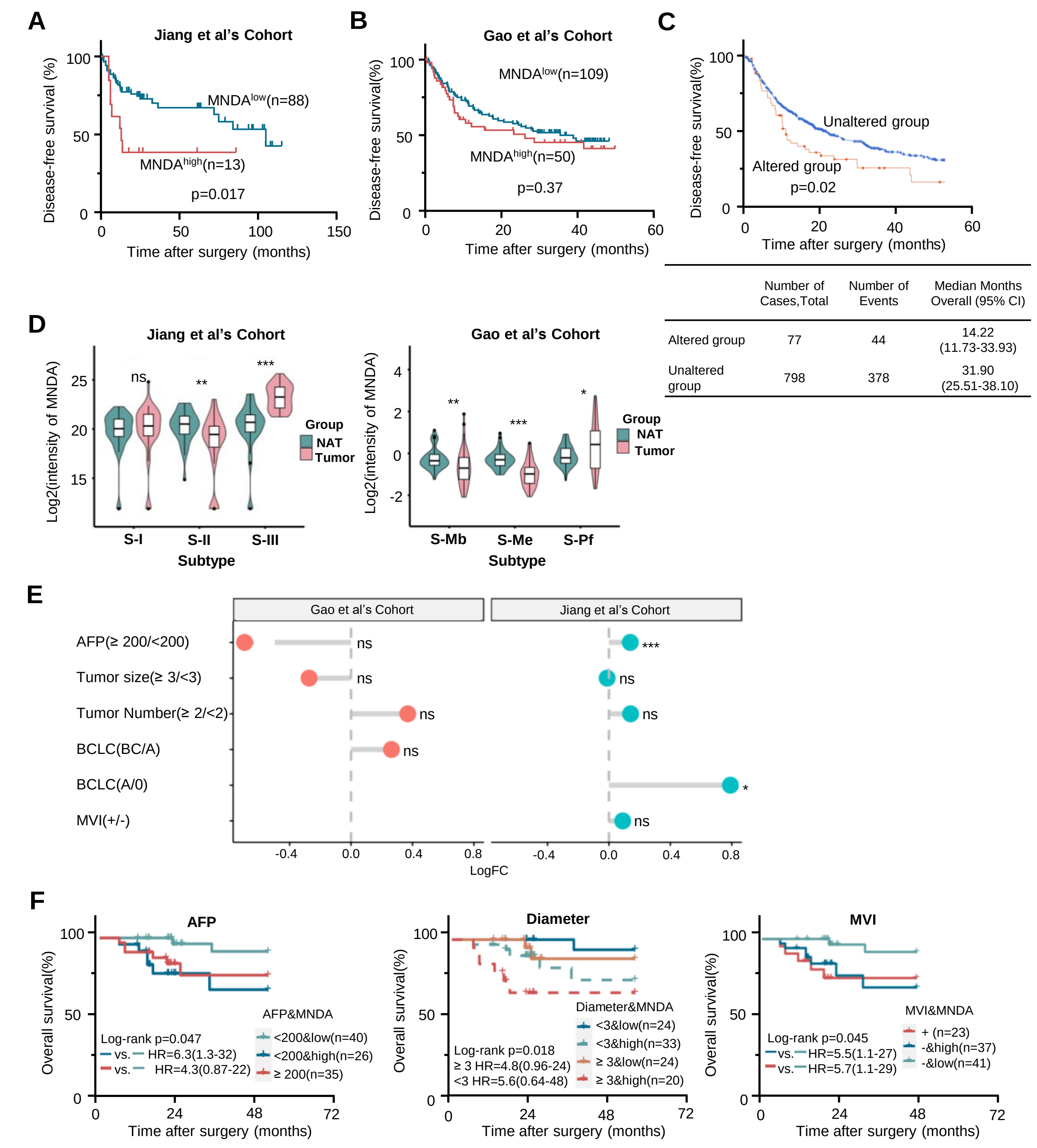

	Number of Cases,Total	Number of Events	Median Months Overall (95% CI)
Altered group	77	44	14.22 (11.73-33.93)
Unaltered group	798	378	31.90 (25.51-38.10)

Supplementary Fig. 2 MNDA augments the prognostic stratification capability of clinical indicators. (A, B) Kaplan–Meier analysis of the DFS probability for HCC patients with different MNDA expression intensities in Jiang et al's cohort, Gao et al's cohort. (C) Kaplan–Meier analysis of the DFS probability for HCC patients with different genomic amplification of MNDA (cBioPortal). (D) Comparison of MNDA protein expression among different subtypes of HCC tissues. (E) Lollipop chart of difference analysis of MNDA expression in AFP (≥200/<200), Tumor size (≥3/<3), Tumor Number (≥2/<2), BCLC (BC/C), BCLC (A/0), MVI (+/-) patients in the Gao and Jiang et al's Cohorts. (F) Kaplan-Meier curves of Jiang et al. 's Cohort demonstrating differences in overall survival among patients with AFP<200 ng/mL & MNDA^{low}, AFP<200 ng/mL & MNDA^{high}, AFP≥200 ng/mL & MNDA; Diameter<3 cm & MNDA^{low}, Diameter<3 cm & MNDA^{high}, Diameter≥3 cm & MNDA^{low}, Diameter≥3 cm & MNDA^{high}; MVI + & MNDA, MVI- & MNDA^{high}, MVI- & MNDA^{low}. (*p<0.05, **p<0.01, ***p<0.001, ****p<0.0001; ns, no significance).

Supplementary Fig. 3

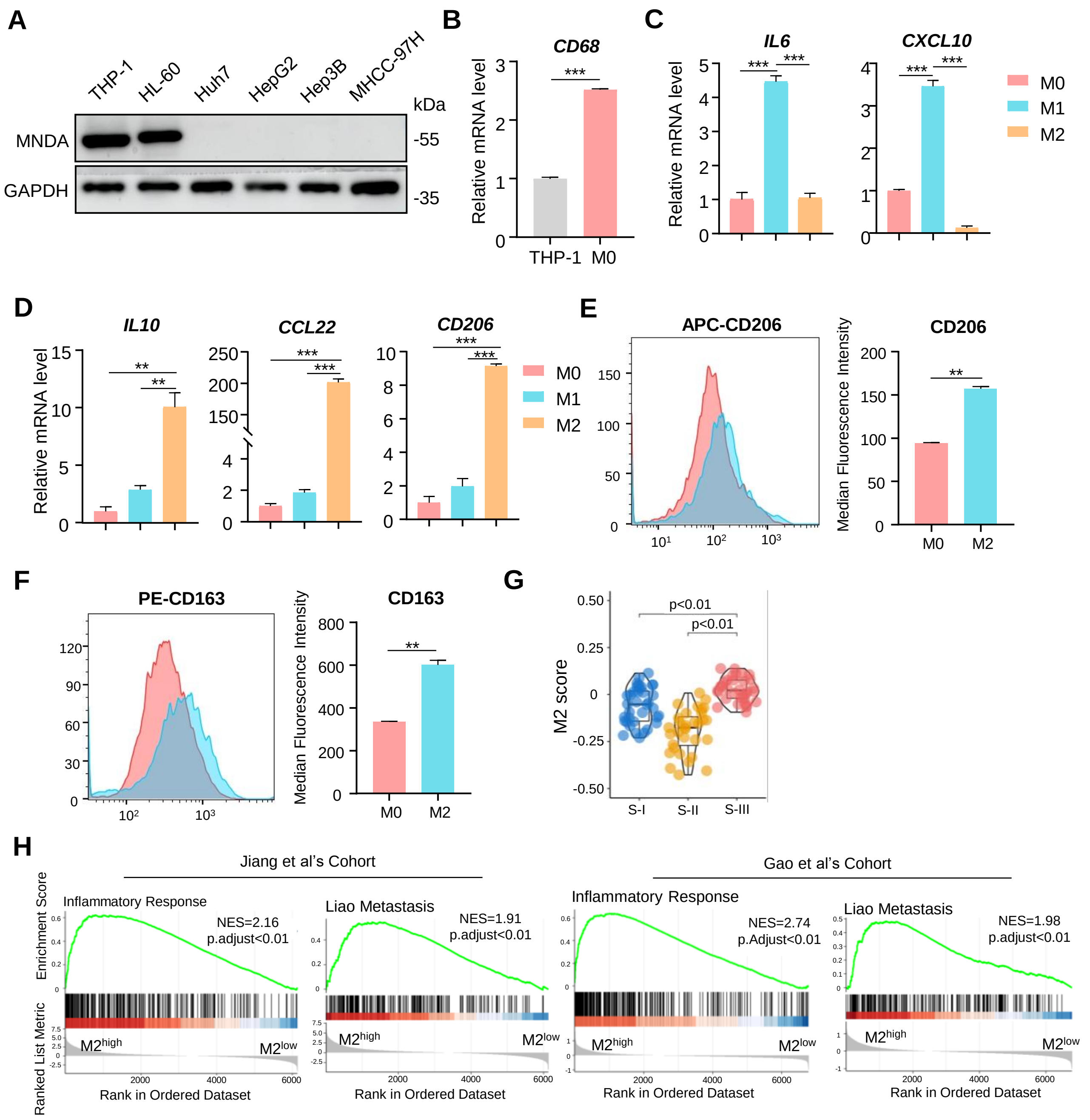

Supplementary Fig. 3 MNDA is mainly localized in M2 macrophages. (A) Western blot analysis of MNDA expression in myeloid cells and liver cancer cells. (B-D) qRT-PCR analysis of M0/M1/M2 macrophage markers. (E, F) Flow cytometry analysis of surface marker expression in M2 macrophages. (G) Comparison of M2 macrophages expression among different subtypes of HCC tissues. (H) Gene set enrichment analysis (GSEA) of inflammatory response and Liao metastasis pathways in the $M2^{low}$ versus $M2^{high}$ groups in two proteome cohorts. Cell experiments were repeated three times independently. (*p<0.05, **p<0.01, ***p<0.001).

Supplementary Fig. 4

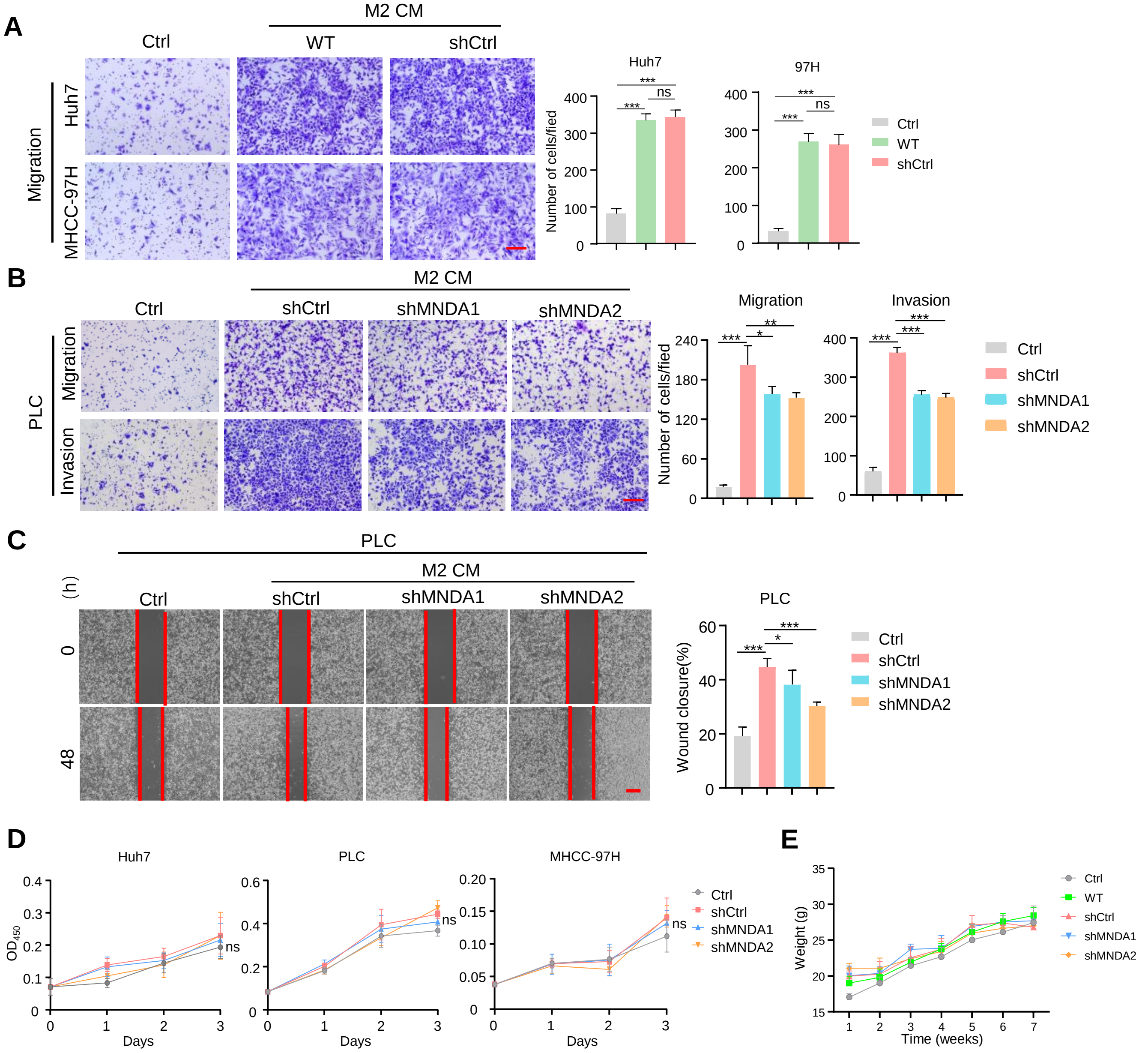

Supplementary Fig. 4 MNDA promotes HCC cells invasion and migration via serum-derived from M2. (A) The migration of Huh7 and MHCC-97H cells after co-culture with Blank, Ctrl, M2 CM-Ctrl, M2 CM-shCtrl were detected by Transwell chamber method. Scale, 200μm. Ctrl: Blank medium; M2 CM-WT: M2-THP-1; M2 CM-shCtrl: M2-THP-1 shCtrl. (B) Transwell chamber assay was used to test cell migration and invasion of PLC cells co-cultured with Ctrl, M2 CM-shCtrl, M2 CM-shMNDA1 and M2 CM-shMNDA2. Scale bar, 200μm. (C) Effects of treatment with Ctrl, M2 CM-shCtrl, M2 CM-shMNDA1 and M2 CM-shMNDA2 on migration of PLC cells, as assessed by wound-healing assay. Images were taken at 0 and 48 hours after scratching (left panel represents representative images; right panel shows percentage of migrated distance quantitatively). Scale bar, 200 μm. (D) Cell proliferation assay of Huh7, PLC and MHCC-MHCC-97H cells co-cultured with Ctrl, M2 CM-shCtrl, M2 CM-shMNDA1 and M2 CM-shMNDA2. (E) Body weight statistics of NOD SCID mice in Fig 6C groups (n=5). Cell experiments were repeated three times independently. (*p<0.05, **p<0.01, ***p<0.001; ns, no significance).

Supplementary Fig. 5

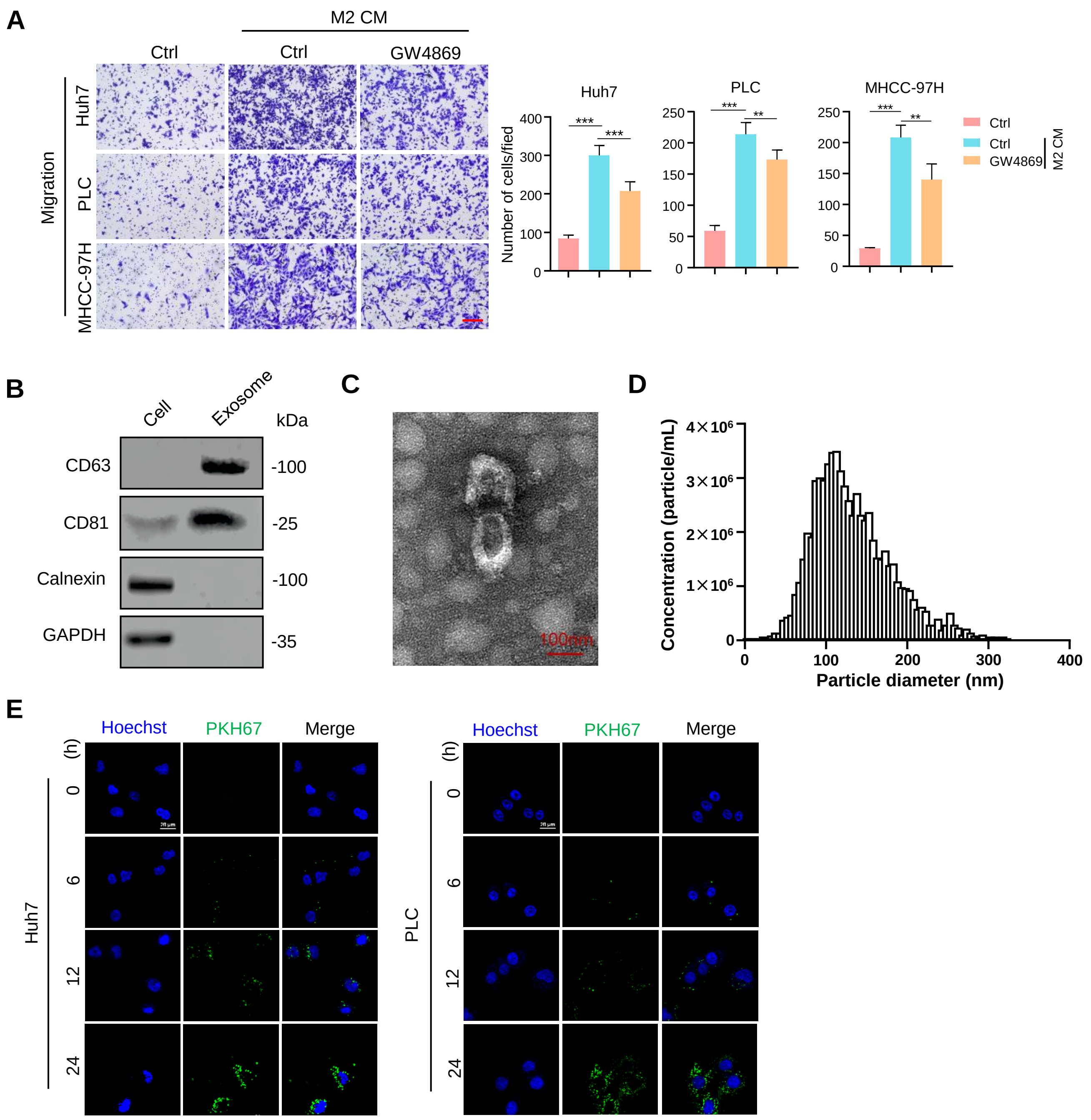

Supplementary Fig. 5 MNDA promotes HCC cells migration via exosome-derived from M2. (A) Effects of treatment with M2 CM and M2 CM + GW4869 on the migration ability of Huh7, PLC and MHCC-MHCC-97H cells, as assessed by a Transwell assay. Representative images are shown in the left panel, and migrated cell counts are shown in the right panel (scale bar, 200 μm). (B) Western blot analysis of the expression levels of CD63, CD81, and calnexin in M2 exosomes. (C) Transmission electron microscope images of the M2 exosomes. Scale bar, 100 nm. (D) The size of exosomes was authenticated by nanoparticle tracking system. (E) Immunofluorescence image of HCC cells co-cultured with PKH67-labeled M2 exosomes, showing uptake of exosomes (green) by HCC cells. Scale bar, 20 μm. Cell experiments were repeated three times independently. (*p<0.05, **p<0.01, ***p<0.001).

Supplementary Table 1. Primer sequences for RT-qPCR

Primers name	Forward sequence (5‘ to 3')	Reverse sequence (5‘ to 3')
CD68	GCTACATGGCGGTGGAGTACAA	ATGATGAGAGGCAGCAAGATGG
IL6	CCACTCACCTCTTCAGAACG	CATCTTTGGAAGGTTCAGGTTG
CCL22	GAGCATGGATCGCCTACAG	CAGACGGTAACGGACGTAATC
CXCL10	CAGACGGTAACGGACGTAATC	ACGTGGACAAAATTGGCTTG
CD206	GCAAAGTGGATTACGTGTCTTG	CTGTTATGTCGCTGGCAAATG
IL10	GCCTAACATGCTTCGAGATC	TGATGTCTGGGTCTTGGTTC
TGFB1	CTCCCGTGGCTTCTAGTGC	GCCTTAGTTTGGACAGGATCTG
ETV1	GCCTATGATCAGAAGCCACAAGT	CCCTTTTCAAACATACAGCCTGAG
MNDA	CCGCAAGAAACAAACTGACA	GATGAGGTCTGGGGTAGTGG
CSF1	AGACCTCGTGCCAAATTACATT	AGGTGTCTCATAGAAAGTTCGGA
BMP7	TTCGTCAACCTCGTGGAACA	CACAGTAGTAGGCGGCGTAG
SGMS2	GATTTGGCAAGATGCTGTGGG	GTGGAGGGCTAAGCTCCTTG
CXCL14	TCCGGTCAGCATGAGGCTCC	AGTCTCTGAATCCTGGCATTG
ITGB5	GTCTGCTAATCCACCCAAAATG	TCTCTATCTCACCTCCACAGC
MMP14	GGCTACAGCAATATGGCTACC	GATGGCCGCTGAGAGTGAC
TIMP1	GGGCTTCACCAAGACCTACA	TGCAGGGGATGGTAAACA
COL6A1	GGGATTCCCTGGACCTAAAG	GGAACACCTCGCTCTCCA
COL6A2	TACGGAGAGTGCTACAAGGTG	GGTCCTGGGAATCCAATGGG
COL6A3	TCTCTTAAAATCAGTGCACAACG	AACTCTTTCAACAGAGGGAAGC
S100A4	ACTGTACATGCTTCGGTCAG	CACCCTATTCTTCGTAGACC
18sRNA	AACTTTCGATGGTAGTCGCCG	CCTTGGATGTGGTAGCCGTTT
ACTB	AGCGAGCATCCCCCAAAGTT	GGGCACGAAGGCTCATCATT

Supplementary Table 2. List of antibodies and reagents used

REAGENT or RESOURCE	SOURCE	IDENTIFIER
	**Antibodies**	
Anti-human MNDA	Sigma	Cat# HPA034532
Anti-human MNDA	Santa	Cat# 390739
Anti-human CD3	Abcam	Cat# 5690
Anti-human CD81	Proteintech	Cat# 66866-1-Ig
Anti-human CD63	Abcam	Cat# 217345
Anti-Calnexin	Abcam	Cat# 22595
Anti-human CD81	Proteintech	Cat# 66866-1-Ig
Anti-human CD163 PE	BioLegend	Cat# 326505
Anti-human CD206 (MMR) APC	BioLegend	Cat# 321109
	**Recombination proteins**	
Human IL-4	Peprotech	Cat# 200-04
Human IFNγ	Peprotech	Cat# 300-02
	**Chemical reagents**	
PMA	Sigma	Cat# P8139
LPS	Sigma	Cat# 28808
GW4869	Selleck	Cat# s7609

Supplementary Table 5. OS and DFS performance of related proteins in two proteomic cohorts

Parameters	OS(p value)	DFS(p value)
**Jiang et.al’s Cohort; Proteomics;n=101**		
TIMP1	<0.001	0.009
NRP2	0.05	0.008
COL6A2	0.027	0.001
COL6A1	0.037	0.024
COL6A3	2.50（0.33-19.14）	0.079
ITGB5	0.03	0.026
ITGAM	<0.001	<0.001
MMP14	0.014	0.038
**Gao et.al’s Cohort; Proteomics;n=159**		
TIMP1	0.003	0.021
NRP2	0.198	0.197
COL6A2	0.019	0.014
COL6A1	0.003	0.025
COL6A3	0.012	0.017
ITGB5	<0.001	0.019
ITGAM	0.021	<0.001
MMP14	<0.001	0.029
